# Exploration of deep terrestrial subsurface microbiome in Late Cretaceous Deccan traps and underlying Archean basement, India

**DOI:** 10.1038/s41598-018-35940-0

**Published:** 2018-11-29

**Authors:** Avishek Dutta, Srimanti Dutta Gupta, Abhishek Gupta, Jayeeta Sarkar, Sukanta Roy, Abhijit Mukherjee, Pinaki Sar

**Affiliations:** 1 Environmental microbiology and genomics laboratory, Department of Biotechnology, Indian Institute of Technology Kharagpur, Kharagpur, 721302 India; 2 School of Bioscience, Indian Institute of Technology Kharagpur, Kharagpur, 721302 India; 3 School of Environmental Science and Engineering, Indian Institute of Technology Kharagpur, Kharagpur, 721302 India; 4 Ministry of Earth Sciences, Borehole Geophysics Research Laboratory, Karad, 415114 India; 5 CSIR-National Geophysical Research Institute, Hyderabad, 500007 India; 6 Department of Geology and Geophysics, Indian Institute of Technology Kharagpur, Kharagpur, 721302 India

## Abstract

Scientific deep drilling at Koyna, western India provides a unique opportunity to explore microbial life within deep biosphere hosted by ~65 Myr old Deccan basalt and Archaean granitic basement. Characteristic low organic carbon content, mafic/felsic nature but distinct trend in sulfate and nitrate concentrations demarcates the basaltic and granitic zones as distinct ecological habitats. Quantitative PCR indicates a depth independent distribution of microorganisms predominated by bacteria. Abundance of *dsr*B and *mcr*A genes are relatively higher (at least one order of magnitude) in basalt compared to granite. Bacterial communities are dominated by *Alpha*-, *Beta*-, *Gammaproteobacteria*, *Actinobacteria* and *Firmicutes*, whereas *Euryarchaeota* is the major archaeal group. Strong correlation among the abundance of autotrophic and heterotrophic taxa is noted. Bacteria known for nitrite, sulfur and hydrogen oxidation represent the autotrophs. Fermentative, nitrate/sulfate reducing and methane metabolising microorganisms represent the heterotrophs. Lack of shared operational taxonomic units and distinct clustering of major taxa indicate possible community isolation. Shotgun metagenomics corroborate that chemolithoautotrophic assimilation of carbon coupled with fermentation and anaerobic respiration drive this deep biosphere. This first report on the geomicrobiology of the subsurface of Deccan traps provides an unprecedented opportunity to understand microbial composition and function in the terrestrial, igneous rock-hosted, deep biosphere.

## Introduction

Microbial life that resides within the deep continental subsurface represents one of the largest and most diverse biospheres on this planet^1^. Recent assessments confirm that in spite of considerable physical as well as chemical constraints, the oligotrophic, dark biosphere underneath the continental crustal system (including fluid-filled pores, and fractures of the igneous rocks) harbour 2–19% of Earth’s total biomass represented by bacteria, archaea and fungi^1–4^. In contrast to areas of abundant resources, deep biosphere within the crustal systems represents an extraordinary environment, as many of the standard forces of ecosystem (e.g. dispersal, metabolic activity and flexibility, thermodynamic feasibility) are either much reduced or not applicable^5^. With mostly unexplored physiological and biochemical details, these microorganisms generally lack culturable phylogenetic neighbours. Interestingly, it remains highly enigmatic that how these vast communities survive in environment that provide only marginal energy for growth, division and minimal metabolic function for cell sustenance^6^.

During the past several decades, our knowledge on geomicrobiology of deep terrestrial subsurface has been broadened significantly^3,7^. It has been established that in contrast to sedimentary rocks, crystalline igneous rocks are typically low in organic matter, porosity and interconnectivity, and have been subjected to high temperature and/or pressure in the geologic past or at present^8,9^. Microbe mediated mineral precipitation may lead to loss of porosity, although secondary increase in porosity at greater depths via pressure dissolution of minerals may also occur^9^. Endolithic microorganisms in such extreme habitats typically occupy the fractures through available interconnections, with nutrients made available either from the rock itself and/or through transportation (via available interconnections) in the form of dissolved gases, solutes, or colloids^3,8,10^. While the lack of photosynthetically derived organic carbon led ‘autotrophy to outstrip heterotrophy’^9^, geogenic supply of CO_2_ and CH_4_ (as carbon source), H_2_, CH_4_, NH_4_^+^ and HS^−^ (as electron donor) and Fe^3+^, Mn^4+^ and CO_2_ (as electron acceptors) have potential role in providing minimal nutrient and energy supply^11^. Hydrogen-driven subsurface lithoautotrophic microbial ecosystems (SLiME) wherein microbial populations can thrive on low concentrations of H_2_ and CO_2_ were initially considered to fuel the subsurface environment^12–15^. Although the dominance of such lithotrophic metabolism has been reported from several terrestrial subsurface including Columbia River basalt^12^, Fennoscandian shield^16^ and Witwatersrand Basin^17^, the role of heterotrophic organisms, capable of utilizing the metabolic products of autotrophs in carbon assimilation and driving the overall community function is highlighted in later studies^18–21^. Overall, a combination of metabolic processes encompassing both autotrophy (including chemolithoautotrophy) and heterotrophy is implicated in deep terrestrial ecosystems^18,20,21^.

Endolithic microbial populations in deep igneous provinces have been investigated in basalt hosted oceanic crusts^22–27^, subsurface crustal environments with high temperature basalts^28^, subsurface gabbros and mantle type rock^29,30^ and crustal fluids^31^. Most of these studies reveal the dominance of bacterial members affiliated to *Proteobacteria* (*Alpha-*, *Gamma-* and *Delta-* sub divisions), *Actinobacteria*, *Firmicutes*, *Bacteroidetes* and *Chloroflexi* known for sulfate reduction, nitrate reduction, fermentation, and chemolithotrophic metabolism^25,26,29,32–34^. Members of archaea, though present in much lower abundance mostly belong to the Miscellaneous Crenarchaeota group (MCG), *Methanosarcinales*, and *Methanobacteriales* taxa. Compared to oceanic system, terrestrial subsurface crustal environments within igneous rocks (basalts and granites) are microbiologically less explored. A few additional studies have investigated the terrestrial subsurface igneous rocks directly^35,36^, while most such studies in the terrestrial realm remain restricted to groundwater samples recovered from basaltic/granitic aquifers^4,35–38^. In contrast to our understanding of structure and function of deep subsurface microbial community through all these studies, the microbiome of Deccan subsurface remains elusive. In this work, we try to demonstrate the nature and function of microbial communities within the deep terrestrial subsurface of Deccan traps using the rock cores recovered through scientific deep drilling^39,40^. Exploration of deep biosphere of granitic-basaltic crustal system is highly imperative to answer the fundamental questions on microbial life within deep, dark, oligotrophic crust, their mode of interaction and role in Earth’s biogeochemical processes, origin of life on Earth and even on extra-planetary locations (e.g., Mars). Compared to a reasonable level of understanding of deep life within marine subsurface, geomicrobiology of subterranean igneous rocks at greater depths remains almost elusive.

Scientific deep drilling in the Koyna-Warna region of Deccan traps, western India provides a unique opportunity to investigate microbial life within deep terrestrial igneous rocks (basalts and granite-gneiss basement)^39,40^. The Deccan traps represents a continental flood basalt province covering an area of over 0.5 million km^2^ of lava flows (~65 Ma) with a total thickness of over 2000 m near the eruptive centre in western India^41–44^. This thick pile of Deccan basalts rests on ~2.5 Ga Archean crystalline basement^45^. The Koyna-Warna region has gained further importance owing to the reservoir triggered seismicity ever since the impoundment of the Koyna dam in 1962^39^. Exploratory core drilling has been carried out at multiple sites in the Koyna seismogenic zone to ascertain the subsurface geology and structure in the region and carry out seismological investigations. The boreholes penetrated the entire thickness of Deccan traps (412–1251 m) and passed through a few hundred meters of the underlying granitic basement^39,46^. Study of drill cores also reveal that the transition of Deccan basalts to granitic basement rock occurs over a relatively short span of few tens of centimetres to few meters^47,51^. In these sections, rocks appear to be intermixed with both granitic and basaltic compositions.

In the present study, rock cores obtained from three exploratory boreholes in the Koyna-Warna region are used to decipher microbial community structure and function. Samples are collected from three horizons, i.e., Deccan basalt (BS) (~65 Ma), the underlying granitic basement rock (GR) (~2500 Ma), and the short section between the two formations representing the weathered surface of granitic basement affected by first lava flows (referred here as a transition zone, TZ). To the best of our knowledge, this is the first report describing subsurface microbiology of the late Cretaceous basaltic lava flows over Archean granitic basement.

## Results

### Geochemical characteristics of the samples

**Figure 1:**
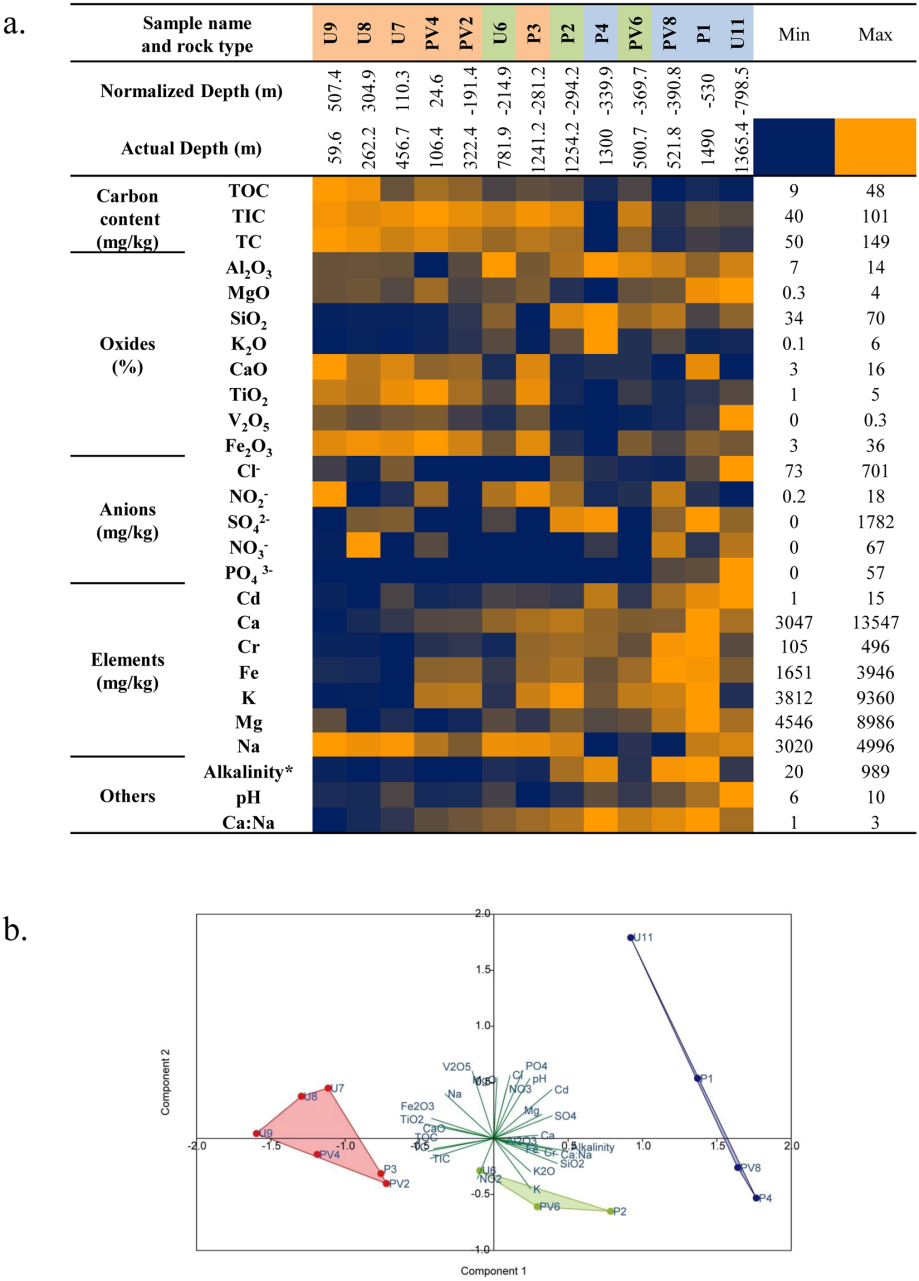
Geochemical characteristics of the samples (**a**) Heatmap showing relative abundances of different measured geochemical parameters. Rock types are depicted in red for basalts, green for transition zone samples and blue for granites; Normalized depth stands for depth from mean sea level (msl) – positive values for samples above msl and negative values for samples below msl. * unit of alkalinity is mg/kg. The samples are arranged according to the increasing normalized depth. (**b**) Principal component analysis of measured geochemical parameters; Rock types are depicted in red for basalts, green for transition zone samples and blue for granites. P: Panchgani, U: Ukhalu and PV: Phansavale.

Thirteen rock core samples covering BS, TZ and GR horizons are collected from three bore holes. Core samples from different depths portray geochemical characteristics of the subsurface system (Fig. 1, Supplementary Tables S1 and S2). Based on the tested parameters we find distinct and characteristic geochemical nature of the granitic and basaltic horizons. Samples from BS zone are mainly composed of feldspar, clinopyroxene (pyroxene mineral), augite (pyroxene mineral), anorthite (calcic plagioclase feldspar), andesine (plagioclase feldspar), hematite (iron bearing mineral), enstatite (pyroxene mineral), diopside (pyroxene mineral), magnetite (iron bearing mineral), magnesioferrite (iron bearing mineral) and albite (plagioclase feldspar). Samples from GR zone show the prevalence of quartz, albite, anorthite, microcline (alkali feldspar), anorthoclase (alkali feldspar) and andesine (plagioclase feldspar). Samples from TZ zone show a mixed nature. Samples from GR zone are more alkaline (pH range 7.8–10.2) and contain higher concentrations of NO_3_^−^, PO_4_^3−^, SO_4_^2−^, Mg, Ca, Fe, K, SiO_2_, Al_2_O_3_ and MgO. BS samples are distinctive based on slightly acidic to neutral pH (pH range 6.4–7.4), relatively higher total organic carbon (TOC) (25.2–48.1 mg/kg), total inorganic carbon (TIC) (93.2–101.2 mg/kg), CaO (14–16% w/w), TiO_2_ (3–5% w/w) and Fe_2_O_3_ (32–36% w/w). Due to the weathered nature of the transition zone, concentrations of a few parameters (e.g., Al_2_O_3,_ K_2_O, NO_2_^−^, K and Fe) do not fit well with the trend observed for granites and basalts. Variations in oxide concentrations with respect to depth in basalts and granites are consistent with the fact that granites are felsic whereas basalts are mafic in nature. Principal component analysis (PCA) based on all the measured parameters shows that the samples from the three different zones (BS, TZ and GR) are geochemically distinctive (Fig. 1b). Analysis of similarities (ANOSIM) further indicate that samples from BS horizon are considerably dissimilar (R = 0.4325, p = 0.026) from GR samples, and TZ samples are more similar (R = −0.2407, p = 0.8856) to GR samples compared to BS samples (R = 0.222, p = 0.1315). The distribution pattern of carbon, nitrogen and phosphorous has been analysed with respect to depth in order to assess the general nutritional condition of the subsurface crustal system. Total organic carbon content shows a strong negative correlation with depth followed by total carbon and total inorganic carbon. Nitrogen (NO_3_^−^and NO_2_^−^) is generally low whereas phosphate is below detection limit except for samples obtained from the deeper horizon (PV8, P1 and U11).

### Sequencing data

Sequence information and diversity indices are summarized in Table 1. 11.5 million reads are obtained for 13 samples. A maximum of 2911866 reads and a minimum of 188953 reads are obtained for samples U11 and P2, respectively. A total of 1012463 reads grouped into 107721 OTUs are finally obtained after quality filtering and control removal. A maximum of 35198 and a minimum of 5148 OTUs are observed in sample U11 and P2, respectively. A maximum of 34080 bacterial and 98 archaeal OTUs are observed in samples U11 and PV2, respectively. A small fraction (0.83–3.67%) of the OTUs remains unassigned across the samples. Considerably high numbers of bacterial OTUs compared to archaeal OTUs are observed across the samples. Noticeably, a total of 71090 unique OTUs (i.e., ~66% of the total number of OTUs) are found across all the 13 samples. These unique OTUs represent a minimum of 22.69% to a maximum of 49.81% of the reads per sample. For comparable alpha diversity analyses, data sets are normalized by random sampling. Chao1 estimator which estimates the minimum total number of OTUs based on the number and size of the OTUs suggest that P3 has the highest and U8 has the lowest OTU diversity. The Shannon diversity index, calculated to evaluate species abundance and evenness in each sample, does not vary much for most of the samples (*H′* 9.26–11.62) with exception of PV2 (3.94) and PV8 (4.87). The Simpson index, which measures the species dominance in a community, lies in a narrow range of 0.98–0.99 for all the samples except PV2 (0.52) and PV8 (0.62). Lower values of Shannon and Simpson indices for samples PV2 and PV8 signify lower diversity in the community when compared to other samples.

### Vertical distribution of microbes

The abundances of bacterial and archaeal 16S rRNA gene copies are determined by quantitative PCR based approach to delineate vertical distribution of microbes (Table 1). Bacterial 16S rRNA gene copy numbers are significantly higher at all depths compared to their archaeal counterparts. Bacterial 16S rRNA gene copies per gram of rock vary between 3.4 × 10^5^ and 4.01 × 10^6^ with an average of 1.61 × 10^6^. Highest bacterial abundance is observed in P2, a sample of weathered granite from 1254 mbsl (meters below surface level). Archaeal 16S rRNA gene copies per gram of rock vary between 1.88 × 10^2^ and 9.41 × 10^4^, with an average of 2.29 × 10^4^. Assuming an average of 4.2 and 1.7 16S rRNA gene copy per genome of bacteria and archaea respectively^48^, our estimate suggests microbial cell abundance ranging from 8.65 × 10^4^ to 1.01 × 10^6^ per gram of rock (average 3.95 × 10^5^; SD = 2.92 × 10^5^). With respect to distribution of estimated bacterial and archaeal cells, higher relative abundance (average of 6.09%; SD = 0.91%) of archaeal cells are observed in samples from the transition zone followed by the basaltic horizon (4.81%; SD = 2.78%). Samples from granitic horizon exhibit on average 1.32% (SD = 1.38%) archaea of the total estimated number of cells. The observed abundance of archaea in samples from TZ and BS zone samples corroborates with higher level of total organic carbon and near constant level of total inorganic carbon in these samples compared to the GR horizon. Abundance of two major biomarker genes (*dsr*B involved in sulfate reduction and *mcr*A involved in methanogenesis) of sulfur and methane metabolism is studied by quantitative PCR across the different rock samples. Presence of *dsr*B gene is noted in all but one sample with copy numbers varying from 7.6 × 10^2^ to 2.7 × 10^5^ per gram of rock with an average of 7.7 × 10^4^ (SD = 6.88 × 10^4^). Gene fragment of *mcr*A is observed in 10 out of 13 samples and its copy number per gram of rock varies from 4.1 × 10^1^ to 2.5 × 10^3^ with an average of 7.7 × 10^2^ (SD = 7.54 × 10^2^). Interestingly, the overall abundances of both the genes are higher by at least one order of magnitude in BS samples when compared with those from TZ and GR.

**Table 1:** Sequencing information and qPCR data.

Sample Name	U9	U8	U7	PV4	PV2	U6	P3	PV6	P4	P2	PV8	P1	U11
Rock type	BS	BS	BS	BS	BS	TZ	BS	TZ	GR	TZ	GR	GR	GR
No. of reads for analysis	25947	20240	211610	167934	132271	97890	16707	20731	15819	11146	72866	13492	205810
No. of archeael OTUs	70	9	97	46	98	54	73	66	21	54	49	82	83
No. of bacterial OTUs	7545	6450	28582	25949	12547	21023	7544	8067	6027	4987	10196	6306	34080
Unassigned OTUs	235	54	877	758	238	698	275	310	192	107	207	155	1035
Percentage of reads unassigned	3.80	0.62	1.12	0.71	0.41	1.50	2.07	3.91	3.26	1.12	0.51	1.47	1.21
% of reads assigned to Archaea	0.80	0.28	0.64	0.16	0.70	0.35	0.99	0.70	0.41	4.98	0.12	6.33	0.24
% of reads assigned to Bacteria	95.40	99.09	98.23	99.13	98.89	98.16	96.94	95.39	96.33	93.90	99.36	92.20	98.54
% reads assigned unique OTUs	38.05	12.72	19.16	15.95	4.98	17.27	35.14	32.61	29.25	22.89	5.82	23.21	18.19
chao1	22162	16779	62595	62604	34761	54564	22860	23240	16919	16702	28177	19284	78727
Shannon’s index	11.22	11.18	11.13	10.82	5.02	11.78	11.88	11.91	11.51	11.14	6.47	11.54	11.51
Simpson’s index	1.00	1.00	0.99	1.00	0.59	1.00	1.00	1.00	1.00	1.00	0.72	1.00	0.99
Goods coverage	0.80	0.79	0.92	0.90	0.93	0.86	0.66	0.72	0.73	0.66	0.90	0.65	0.90
Observed OTUs^#^	2898	2649	2487	2657	3320	3172	6205	3170	3995	2981	2642	5097	2524
chao1^#^	7135	6400	6666	7854	9746	7889	17175	7084	11758	7897	6891	13613	7718
Shannon’s index^#^	10.30	9.97	9.27	9.26	3.94	10.45	11.62	10.68	10.67	10.69	4.87	11.01	9.50
Simpson’s index^#^	1.00	1.00	0.99	0.99	0.52	1.00	1.00	1.00	1.00	1.00	0.62	1.00	0.98
Estimated bacterial cells	7.3E + 05	4.2E + 05	1.8E + 05	ND	8.1E + 04	1.1E + 05	3.2E + 05	2.1E + 05	5.2E + 05	9.6E + 05	1.1E + 05	2.8E + 05	6.8E + 05
Estimated archaeal cells	1.3E + 03	2.8E + 04	1.4E + 04	ND	5.4E + 03	8.0E + 03	1.4E + 04	ND	1.1E + 02	5.5E + 04	2.9E + 03	5.7E + 02	1.8E + 04
*dsr*B copy number	6.4E + 04	2.7E + 05	1.2E + 05	ND	5.4E + 04	6.6E + 04	1.0E + 05	7.6E + 02	7.2E + 04	8.6E + 04	5.3E + 04	3.1E + 04	1.4E + 04
*mcr*A copy number	4.1E + 01	1.6E + 03	9.8E + 02	ND	2.1E + 02	4.1E + 02	2.5E + 03	ND	ND	8.5E + 02	2.5E + 02	3.8E + 02	4.8E + 02

ND- not detected; all the estimated cell numbers and gene copy numbers are measured per gram of rock. Samples from basalt, transition and granitic zones are designated as BS, TZ and GR, respectively. ^#^Depicts subsampled alpha diversity data.

### Microbial community analysis

**Figure 2:**
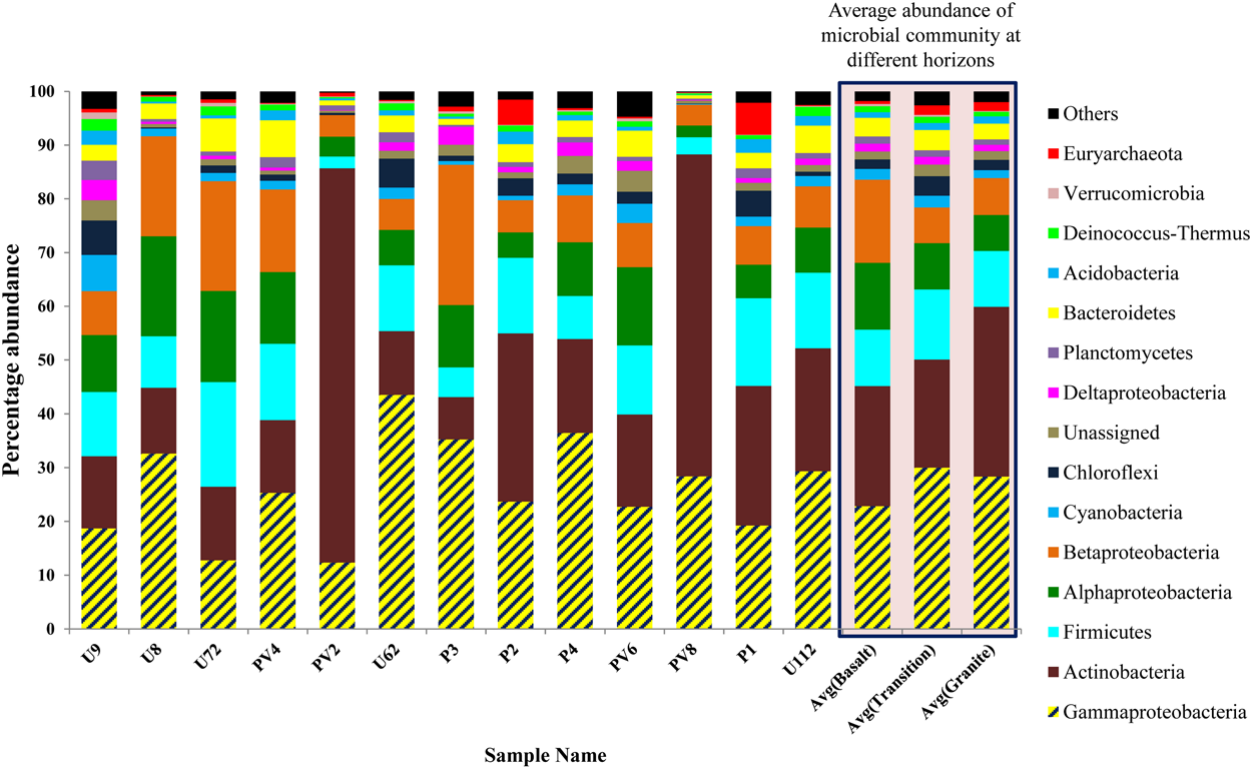
Microbial community composition of Deccan subsurface (at phylum/class level). Phyla/classes which have minimum 1% abundance in one of the 13 samples were selected and the remaining ones are grouped into ‘Others’.

Sequences assigned to bacteria cover 54 phyla, 127 classes, 212 orders, 377 families, and 1049 genera whereas sequences assigned to archaea cover 12 phyla, 12 classes, 8 orders, 15 families, and 23 genera. On the basis of average relative abundance analysis at phylum level, *Proteobacteria*, *Actinobacteria* and *Firmicutes* constitute the three major populations of the deep biosphere of Deccan trap region with average relative abundance >11% (Fig. 2). Among those, *Proteobacteria* is the most dominant (20–77%) followed by *Actinobacteria* (8–73%) and *Firmicutes* (2–19%) (Fig. 2). *Bacteroidetes*, *Chloroflexi*, *Cyanobacteria*, *Planctomycetes*, *Acidobacteria* and *Deinococcus-Thermus* are the other phyla constituting the communities, with >1% average relative abundance across the samples, but often much higher levels in specific samples/type of rocks. *Euryarchaeota* is the most dominant archaeal phylum detected across all the samples followed by *Thaumarchaeota* whose presence is observed in all but PV8 and U7 samples.

On the basis of cumulative abundance, *Gammaproteobacteria*, *Actinobacteria*, *Betaproteobacteria*, *Alphaproteobacteria* and *Bacilli* are identified as the five major bacterial classes. Average relative abundance of *Gammaproteobacteria* in the BS cores is lower (23%; SD = 10%) than the GR (28%; SD = 12%) and TZ (30%; SD = 7%) cores. On the other hand, average abundance of *Alpha-* and *Beta-Proteobacteria* are higher in the BS cores (12% and 15% respectively; SD of 5% and 8% respectively) than that of GR (~7% for both; SD of 3% and 2% respectively) and TZ (9% and 7% respectively; SD of 5% and 1% respectively). Out of 12 assigned archaeal classes, *Thermoplasmata* exhibits highest average abundance followed by *Methanobacteria*, *Methanomicrobia* and *Halobacteria*.

Taxonomic affiliation at the lowest level (genus) is possible only for 89% of total classified reads (91% of the OTUs). *Acinetobacter* followed by *Corynebacterium*, *Pseudomonas* and *Staphylococcus* represent the most abundantly detected genera. Other bacterial genera which are detected in significant amount across all the samples are *Enhydrobacter*, *Halomonas*, *Micromonospora*, *Rhizobium*, *Agromyces*, *Bacillus*, *Sphingomonas*, *Shewanella* and *Deinococcus*. On the other hand, at genus level, *Thermoplasma*, *Methanobacterium* and *Methanosaeta* are found to be the most abundant archaeal genera.

### Correlation among different microbial classes

**Figure 3:**
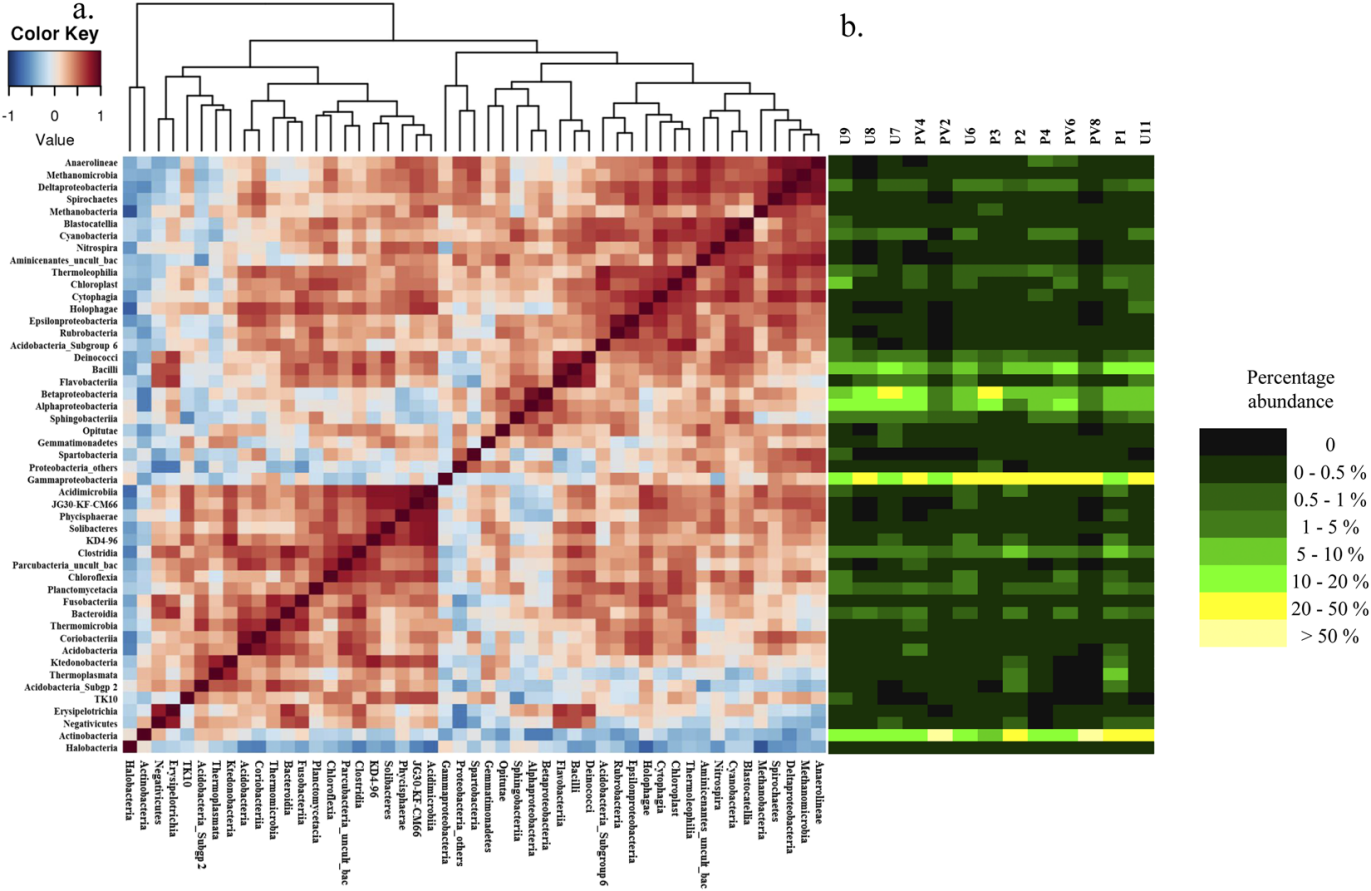
(**a**) Heatmap of Spearman correlation based on abundance across microbial classes having greater than 0.1% average relative abundance across the samples. (**b**) Heatmap of relative percentage abundance of corresponding microbial classes across all the samples.

Spearman correlation is used to identify the populations (at class level) whose abundances are strongly correlated (Fig. 3). A number of classes with relatively lower abundance, *viz*., *Anaerolineae*, *Methanomicrobia*, *Deltaproteobacteria*, *Spirochaetes*, *Methanobacteria*, *Blastocatellia*, *Cyanobacteria* and *Nitrospira* show strong positive correlation among one another. *Thermophila*, *Chloroplast*, *Cytophagia*, *Holophagae*, *Epsilonproteobacteria*, *Rubrobacteria* and *Acidobacteria* subgroup 6 form the second distinct clade. *Deinococci*, *Bacilli* and *Flavobacteria* also have strong correlation among each other. Relative abundances of *Acidimicrobiia*, JG30-KF-CM66, *Phycisphaerae*, *Solibacteres*, KD4–96, *Clostridia*, *Parcubacteria_uncult_bac*, *Chloroflexia*, *Planctomycetacia*, *Fusobacteriia*, *Bacteroidia*, *Thermomicrobia*, *Coriobacteriia* and *Acidobacteria* are well correlated mutually. Relatively more abundant classes like *Gammaproteobacteria*, *Actinobacteria*, *Beta-* and *Alphaproteobacteria* show poor correlation among one another.

### Similarity among the bacterial communities

**Figure 4:**
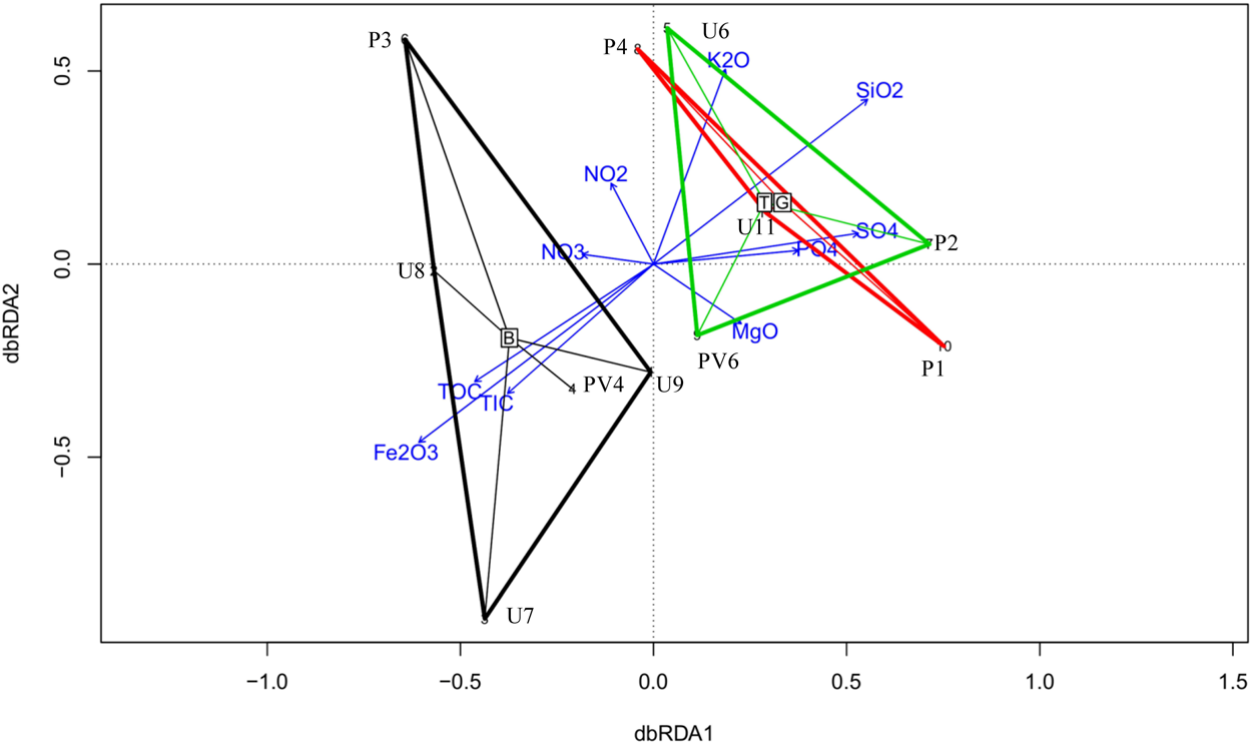
Distance-based redundancy analysis (db-RDA) on the basis of Bray-Curtis distance of microbial communities and their association with environmental factors of the rock cores of Koyna-Warna region. Black triangle represents clusters of samples from BS horizon; Green triangle represents clusters of samples from TZ horizon; Red triangle represents clusters of samples from GR horizon.

Relationships between microbial community structures at different horizons (BS, TZ, GR) and environmental parameters have been established. Distance based redundancy analysis (db-RDA) separate the samples into two distinct abundance-weighted categories. Regardless of the borehole sites, subsurface rock microbiomes partition into (i) BS zone communities and (ii) TZ-GR zone communities (Fig. 4). Average taxonomic distribution patterns demonstrate that compared to both TZ and GR or only GR, BS microbiomes have relatively greater relative abundance of *Alpha-* and *Betaproteobacteria* and lesser abundance of *Gammaproteobacteria*, *Actinobacteria*, *Chloroflexi* and *Euryarchaeota*. GR microbiome in this reference show elevated dominance of *Actinobacteria* compared to its BS and TZ counterparts. SIMPER analysis is used to determine the microbial taxa (class level), responsible for the differences observed between the BS, TZ and GR microbiomes (Table 2). Additionally, this data also demonstrates the groups responsible for the overlap of transition zone and granitic horizon microbiomes. The top most ‘discriminating’ taxa are *Gammaproteobacteria*, *Actinobacteria*, *Beta*- and *Alphaproteobacteria* followed by *Bacilli*, *Thermoplasmata*, etc.

**Table 2:** SIMPER analysis result displaying top 10 classes responsible for dissimilarity among basalt, granite and transition zone.

Taxon	Av. dissim	Contrib. (%)	Cumulative %	Mean Basalt	Mean Transition	Mean Granite
*Gammaproteobacteria*	5.167	17.4	17.4	24.9	30	28.3
*Actinobacteria*	4.277	14.41	31.81	10.6	18.1	20
*Betaproteobacteria*	4.235	14.26	46.07	17.8	6.64	7.87
*Alphaproteobacteria*	2.887	9.723	55.79	14.2	8.65	8.2
*Bacilli*	1.529	5.149	60.94	9.09	9.19	9.13
*Thermoplasmata*	0.9665	3.255	64.2	0.162	1.54	2.01
*Clostridia*	0.7181	2.419	66.62	2.52	3.21	3.15
*Sphingobacteriia*	0.6415	2.161	68.78	1.97	1.1	1.31
Unassigned	0.6361	2.142	70.92	1.66	2.18	1.98
*Deltaproteobacteria*	0.6126	2.063	72.98	1.76	1.44	1.55

### Endemic Deccan subsurface microbiome and OTU level analysis

Core OTUs, i.e., those present in all samples of a particular rock type from each of the three geologic horizons BS, TZ and GR are analyzed. Overall, 131, 337 and 143 OTUs constitute the core OTUs of BS, TZ and GR microbiomes respectively (Supplementary Fig. S1). Among those OTUs, a considerable proportion (74%, 84% and 66% respectively) is unique to each geologic horizon and only 15 OTUs are found to be shared among all the 13 samples (Supplementary Fig. S2). Taxonomic distribution of the core OTUs present a considerable difference among the three zones (Supplementary Fig. S3). Compared to GR microbiome, BS exhibits relatively higher abundance of *Actinobacteria* (*Corynebacteriales* and *Propionobacteriales*), *Alphaproteobacteria* (*Rhizobiales*, *Sphingobacteriales*, *Rhodobacteriales*) and *Betaproteobacteria* (*Burkholderiales*), but lower abundance of *Bacilli* and *Gammaproteobacteria* (*Pseudomonadales*, *Enterobacteriales*, *Oceanospirillales* and others). TZ microbiome core community is more enriched. *Actinobacteria*, *Alphaproteobacteria* and *Bacilli* are mainly present with elevated abundance. However, lower abundance of *Gammaproteobacteria*, and unique presence of *Clostridia*, *Bacteroidia*, *Deltaproteobacteria*, and *Thermoleophilia* are noted. Marked difference is noted with respect to *Betaproteobacteria* in BS and GR horizons. Compared to BS, wherein *Betaproteobacteria* is solely represented by *Burkholderiales*, at least three more highly relevant orders, *viz*., *Hydrogenophilales*, *Methylophilales* and *Nitrosomonadales* are present in GR (as *Betaproteobacteria*). Rank abundance histogram for these shared OTUs across the three different rock microbiomes mostly indicates uneven distributions (Supplementary Fig. S4). These shared OTUs belong to the members of *Gammaproteobacteria*, *Betaproteobacteria*, *Actinobacteria*, *Deinococci* and *Sphingobacteriia* and among these, *Idiomarina*, *Nocardioides*, *Mesorizobium*, unclassified *Chitinophagaceae* and *Delftia* represent the top 5 OTUs.

### Microbial co-occurrence network analysis

**Figure 5:**
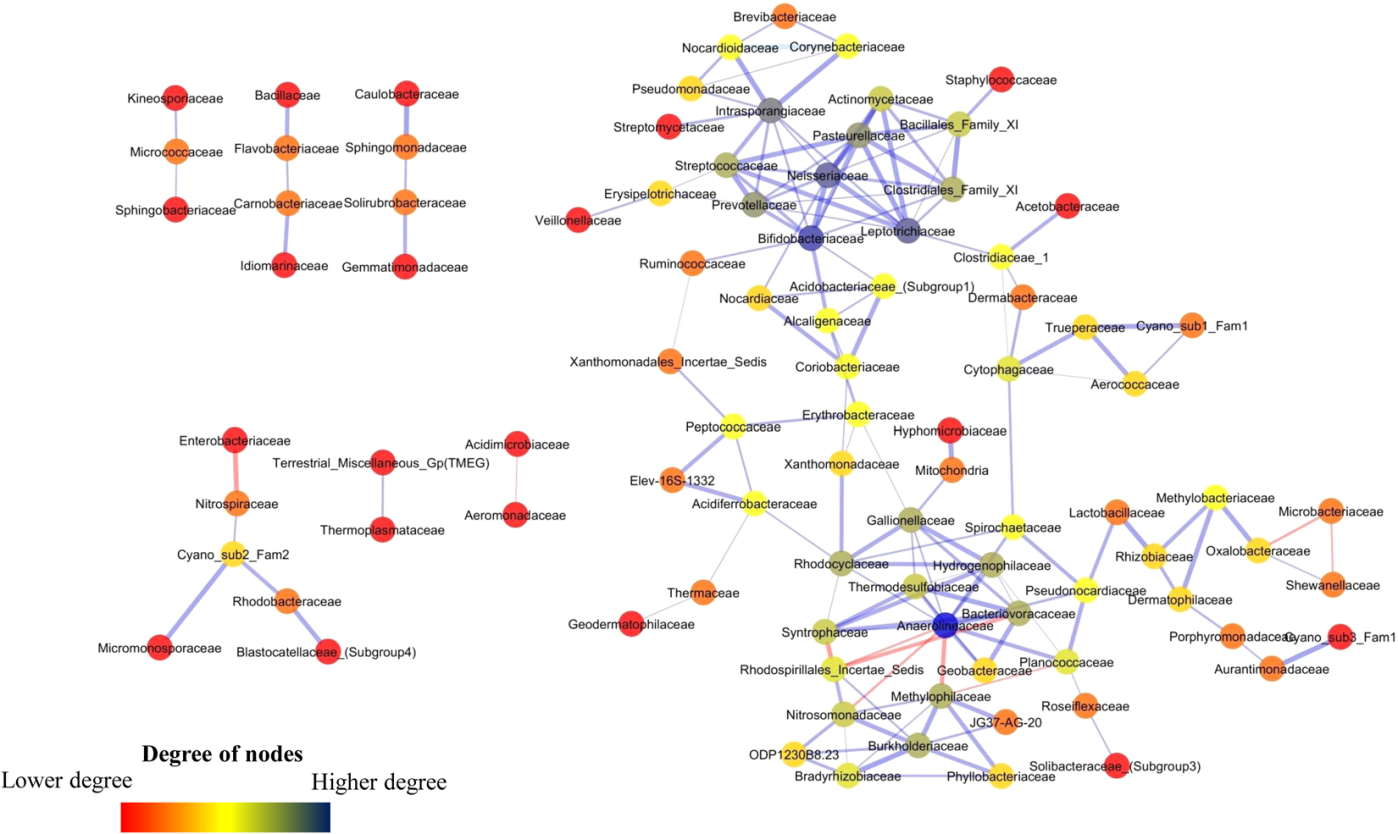
Co-occurrence network of microbial community (family level) in subsurface rock cores of Koyna-Warna region. The colour of the nodes represents the number of degrees. Higher the degree of a node, the bluer the colour of the node; lower the degree of a node, the redder the colour.

Microbial co-occurrence network analysis is performed based on family level data obtained across the three horizons. Network consists of different sub-networks which are composed of both positive and negative connections on the basis of Spearman correlation. The main network consisting of 82 families has two distinct hotspots (or regions where large number of connections are observed among nodes) (Fig. 5). One of the hotspots (designated as hotspot 1) mainly constitutes *Bacillales* Family XI, *Clostridiales* Family XI, *Neisseriaceae*, *Pasteurellaceae*, *Bifidobacteriaceae* and *Prevotellaceae*. The second hotspot mainly includes groups like *Anaerolineaceae*, *Hydrogenophilaceae*, *Syntrophaceae*, *Bacteriovoracaceae*, *Rhodocyclaceae*, *Rhodospirillales*, *Nitrosomonadaceae* and *Gallionellaceae*. In addition to this, several micro-networks that are devoid of any connections to the main network are also observed. The members of micro-network 1 consist of *Nitrospiraceae* and others whereas micro-network 2 harbours two archaeal groups (*Thermoplasmataceae and* Terrestrial Miscellaneous Group).

### Metagenomic inventory

**Figure 6:**
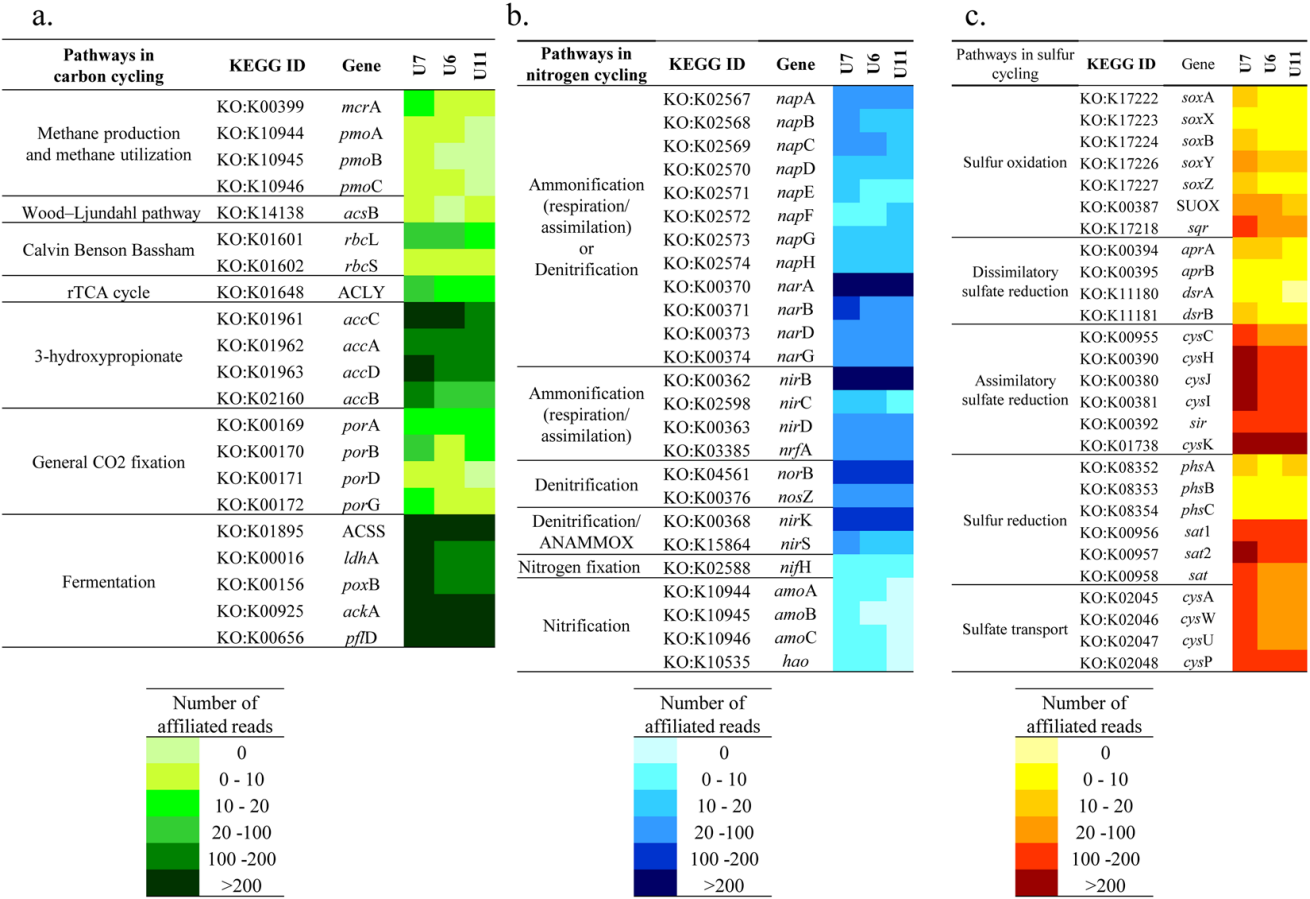
Abundance of affiliated reads related to different pathways of (**a**) carbon (**b**) nitrogen and (**c**) sulfur cycle across three different representative samples from different horizons of Deccan subsurface.

Metagenomic inventories of three representative rock microbiomes from BS, TZ and GR zones are studied through shotgun metagenomics approach. Metagenomic sequencing information and assembly statistics are provided in Supplementary Table S3. After assembling the metagenomic reads, around 3 million contigs having varying length are obtained for each sample using K-mer of 55. Size of the largest contigs varies between 0.3–0.4 Mb. Taxonomy and distribution of different genes are investigated. Among the prokaryotic members, bacterial gene abundance is found to be considerably higher compared to archaeal gene abundance (on the basis of COG-based hits having more than 30% identity) (Supplementary Table S3). Metagenomic data is analysed to gain a first insight into the factors that allow sustenance of observed microbial communities, particularly the interactions among community members and their contribution in carbon, nitrogen and sulfur cycles in the nutrient-limited igneous subsurface of Deccan traps. Genes involved in carbon cycling pathways mainly in carbon fixation *viz*. *rbc*L, *acs*B, *acc*C and ACLY, methane production (*mcr*A), methane utilization (*pmo*A) and different fermentative processes (ACSS, *ldh*A, *pox*B, *pfl*D, *ack*A and others) have been detected (Fig. 6a and Supplementary Fig. S5a). Key genes coding for CO_2_ fixation by Calvin-Benson-Bassham cycle (*rbc*L), 3-hydroxypropionate cycle (*acc*C) and reverse TCA cycle (ACLY) are observed in all the samples with varying abundance whereas the gene responsible for CO_2_ fixation in Wood–Ljungdahl pathway (*acs*B) is observed in U7 (BS) and U11 (GR) only. Gene involved in methane production, *mcr*A is detected in all the samples whereas gene involved in methane utilization, *pmo*A is detected in BS and TZ zone samples only. Overall abundance of affiliated reads to *mcr* and *pmo* are considerably lower than the reads affiliated to marker genes of different carbon fixation pathways. 3-hydroxypropionate pathway is found to be the most dominant carbon fixation pathway in the Deccan subsurface (Fig. 6a). Presence of different marker genes taking part in various nitrogen cycling pathways is detected in all the three samples. Evidence of denitrification (*nar*, *nap*, *nir* and *nor*) is found. Presence of both cytoplasmic (*nar*ABDG) and periplasmic (*nap*ABC) nitrate reductases and all other enzymes that perform steps in the complete denitrification process suggests that dissimilatory nitrogen-transforming metabolisms are common (Fig. 6b and Supplementary Fig. S5b). Furthermore, presence of nitrogen fixation gene (*nif*H) is observed in all the metagenomes whereas genes aiding in the process of nitrification (*amo*A and *hao*) are observed in U7 (BS) and U6 (TZ). Sulfur cycle is one of the important processes that take place in the subsurface environment where microorganisms are a significant contributor. Presence of genes taking part in dissimilatory sulfur reduction (*dsr* and *aps*AB), assimilatory sulfur reduction (*cys*C, *cys*H, *cys*JI and *sir*) and sulfur oxidation (*sqr*, *sox* and SUOX) are detected in the subsurface metagenomes (Fig. 6c and Supplementary Fig. S5c). However, in Deccan subsurface, the number of reads affiliated to dissimilatory sulfur reduction genes is lower than genes involved in assimilatory sulfur reduction and sulfur oxidation (Fig. 6c). Presence of genes responsible for hydrogenase activity (*hyp*ABCDEF) is also observed in low and varied abundances across the three samples.

## Discussion

The subsurface rock samples from the Koyna-Warna region in western India span a substantial geological timescale, varying from ~65 Ma (Deccan flood basalt) to Archaean (basement granitoids) and including a small zone of weathered basement rocks that experienced the first rounds of lava flows during the emplacement of the Deccan traps^44,45,49^. Geochemical attributes of the subsurface rock samples suggest distinct partitioning of the three major crustal provinces (BS, TZ and GR) deep underneath the Deccan traps. During last five decades, the Koyna-Warna region has experienced recurrent seismicity attributable to the changing water levels in the Koyna reservoir, potentially causing structural deformation and associated fault and fracture zones in the adjoining regions^47,50–52^. The samples from all three zones used in this study portray characteristic geochemical signatures with respect to minerals present, alkalinity, and low organic carbon along with distinct pattern of abundance for oxides. Nevertheless, variations as evident in a few samples could be attributed to geological phenomena. The variations are mostly in terms of relative abundance of oxides, in particular, higher weight percentage of Fe_2_O_3_ and lower weight percentage of SiO_2_ than the standard basalts and granites^53^. Further investigation on rock mineralogy by our laboratory (data not shown) indicates that apart from the characteristic minerals of igneous rocks (like pyroxenes, plagioclase feldspar and alkali feldspar)^54^, some samples contain metasomatic minerals (like berlinite, dravite, pyrochlore and phlogopite). Presence of these metasomatic minerals indicates secondary alterations and possible movement of fluids through different faults and fractures. These results are consistent with a previous study which depicts structural deformations and several instances of water movement through different fractures and faults associated with seismicity in the Koyna-Warna region^51^.

Microbial life within the nearly impermeable, highly oligotrophic basalts and granitic bedrock is presumed to be constrained by prevailing physical and chemical factors. The igneous rocks in the deep subsurface have very low porosity (<0.2 ± 0.2% for granitoids)^50^ and moderate-to-high temperature and pressure (25 °C increase per km in basalt and 15 °C increase per km in granite; 26.7 MPa increase in lithostatic pressure per km)^55^. In comparison to several other subsurface igneous provinces, the BS, TZ and GR samples of the present study show significantly low (over one order of magnitude) organic carbon (TOC)^56–58^. Deep subsurface igneous provinces are often devoid of photosynthetically derived organic carbon. Nevertheless, the geogenic carbon (in the form of dissolved inorganic carbon, organic acid, abiogenic CH_4_, C_2–4_ hydrocarbons), electron donors (CO_2_, CH_4_, H_2_, reduced -S and -N) and electron acceptors (NO_3_^−^, NO_2_^−^, SO_4_^2−^, Fe^3+^, Mn^4+^, etc.) can act as essential metabolic resources and facilitate microbial life^59^. From the overall geochemical data, we could elucidate the nutritional status of this igneous realm which suggests its potential to support microorganisms that prefer chemolithotrophic/chemolithoautotrophic lifestyle. The chemolithotrophic/chemolithoautotrophic mode could be linked to heterotrophic metabolism through metabolic products of lithotrophic organisms, thus driving the community’s overall function.

Our qPCR results on 16S rRNA gene abundance indicate the presence of nearly 10^5^ cells per gram of rock. Bacteria outnumber archaea in all samples. Estimation of cell numbers from qPCR based gene copies is done considering an average 16S gene copy per genome and this estimate may be prone to bias^27^. Additional caveat may be due to primer bias during 16S rRNA gene amplicon preparation. Nevertheless, the 16S rRNA gene amplicon data, qPCR based analysis and whole metagenome based data corroborate among each other with respect to microbial community composition. Cell number observed in our study is consistent with the “rocky deep biosphere trend”^9^. Current estimated cell number in various deep subsurface igneous provinces range between 10^4^ and 10^8^ cells per gram of rock^27,60–64^. Lack of correlation of cell number with depth within deep subsurface of Deccan traps is consistent with previous reports on several other terrestrial deep subsurface studies^33,34,65^. Relative consistency of cell numbers across depth in Deccan traps could be attributed to a common set of metabolic or ecophysiological constraints to the inhabiting cells. Considering the geologic age of formations and very low porosity (<0.2 ± 0.2%) of the rocks^50^, the origin of the observed subsurface basaltic and granitic crustal communities remains enigmatic. The only plausible explanation is dispersion of cells through water movement in various fractures and faults developed due to tectonic perturbations. A recent study suggests movement of water through fissures and fractures in the Deccan subsurface of Koyna seismic zone which is evident from secondary mineralization in basalts and basement granitoids^51^.

16S rRNA gene based community composition corroborates with our qPCR based estimation suggesting a more diverse bacterial population when compared to archaea. Considerably higher diversity of bacteria as observed in this study is in the same order of magnitude or even higher than those reported from other deep terrestrial habitats from a similar depth range^16,35–37^. Sequencing depth attained in our work is significant leading to the detection of more OTUs and diversity. In addition to this methodological aspect (sequencing depth), previous studies have linked higher diversity in igneous rocks with the rock age and thus its alteration state. Younger basalts are reported to host less diverse communities while older basalts (>20000 years) generally display richer communities adapted to local alterations of the rocks^24^.

Phylum level composition of microbial communities of deep Deccan subsurface corroborates with recent surveys on microbial life within igneous rocks (basalts, granites and volcanic glasses) indicating predominance of *Gamma-* and *Alpha/Betaproteobacteria*, *Actinobacteria*, *Chloroflexi*, *Firmicutes*, *Nitrospirae*, *Deltaporteobacteria*, and *Epsilonproteobacteria*^22,38,61,66–69^. The global occurrence of most of these taxa in almost all basaltic-granitic environments could possibly be considered as an indication for igneous rock hosted core group^27^. Members of *Alpha-* and *Betaproteobacteria* are relatively more abundant in BS horizon. Most of these proteobacterial taxa are known to be of diverse metabolic types especially H_2_/Fe^2+^/S oxidizing autotrophs; C1 compound-, hydrocarbon- metabolizing, microaerophilic, fermentative and strict anaerobic organotrophs^70^. *Actinobacteria*, though found in all three zones, is relatively more abundant in GR. Among the actinobacterial classes, presence of obligate acidophilic *Acidimicrobiales* members commonly found in Fe-rich ecosystem (including sites with volcanic rocks) and with Fe^2+^ oxidizing or Fe^3+^ reducing abilities justifies their niche specific colonization within the deep subsurface of Deccan traps^71^. A small proportion (average abundance ~2%; SD = 1.7%) of the communities is represented by *Cyanobacteria*. Despite that the ‘rocks at this site have not seen sunlight’ for several thousands to millions of years, presence of *Cyanobacteria* in the deep subsurface of Deccan traps environment corroborates well with several earlier deep biosphere studies, indicating their potential role in supporting sulfate reducing bacteria by providing dark fermentation derived H_2_^72–75^. *Cyanobacteria* are considered to be ubiquitous in aquatic environments and it is possible that a cyanobacterial bloom at or near surface was trapped in the aquifer long time ago and/or the water might have intruded into the deeper layers of rock strata through faults/fractures. Whether these phototrophs survived because of their ability to switch to a fermentative lifestyle^76^ or they are less prone to degradation cannot be determined with the data in hand and remains an open question to answer through further research.

Correlation among the microbial classes based on their abundance highlights the possible interplay among the groups. On the basis of known metabolic properties of these interrelated taxa, we hypothesize a possible metabolic roadmap followed by the Deccan deep subsurface microbiome which allows utilization of available resources and provides survival benefits. Close association of several taxa previously known from diverse deep, oligotrophic igneous subsurface and capable of autotrophic carbon fixation (*Anaerolinea*, *Cyanobacteria*, *Epsilonproteobacteria*), lithotrophic nutrition with oxidation of inorganic nitrogen (NO_2_^−^), sulfur (S^0^) and iron (Fe^2+^) (*Spirochitae*, *Nitrospira*, *Acidimicrobia*) and organotrophic nutrition with fermentative, denitrifying and sulfate reducing activities (*Firmicutes*, *Actinobacteria*, *Deltaproteobacteria*) can be noted^21,77–84^. Presence of methanogenic archaea (*Methanomicrobia*, *Methanobacteria*) along with *Deltaproteobacteria* is a clear indication of interspecies metabolite transfer through syntrophic activities. Close metabolic tie-up between autotrophic and heterotrophic members of the communities is previously reported from several oligotrophic deep subsurface ecosystems^20,21,75,77^. Although present in low abundance, correlation among *Nitrospira*, *Spirochaetes*, *Chloroflexi*, *Cyanobacteria* and *Epsilonproteobacteria* indicates that they could be the key players in driving the redox reactions and carbon flux through oxidation of inorganic substrates (e.g., reduced sulfur, nitrite, hydrogen) and dark fixation of carbon in the nutrient deprived, mineral rich, deep, dark Deccan subsurface^82–84^. Assimilated carbon and its fermentation products (*e*.*g*. H_2_, acetate, formate, etc.) drive other lithotrophic and heterotrophic metabolism including anaerobic nitrate/sulfate reduction and methanogenesis. Lower level of correlation among more abundant proteobacterial groups as evident from our study has been previously noted in the Fennoscandian shield, and could support the hypothesis that close interrelations among these taxa are possibly more relevant on or near surface rather than in deep terrestrial subsurface^16^.

It is interesting to note that a high percentage of OTUs are unique to BS, TZ and GR microbiomes (as sampled from various depths) and only a handful of OTUs (15/107721) covering 2.8% of the total sequences are shared among all the 13 samples. This possibly indicates community isolation between different horizons (lava flows in basalt, transition zone and granitic province). This particular observation presents a sharp contrast to the high degree of overlap in OTU distribution pattern in deeply buried oceanic crust and sedimentary habitat above^26^. Interestingly, the observed partitioning of BS and GR microbiomes (as observed in db-RDA) corroborates well with the previous reports of a basalt specific microbiome^85,86^. Based on these results, we propose that although taxonomically similar organisms have occupied different igneous rocks at varied depths, environment specific taxon recruitment based on local availability of electron donors, acceptors and carbon sources, and limited opportunities of migration led to the distinct taxonomic assemblages. Our db-RDA data clearly indicate such partitioning of interrelated guilds and these distinct microbial assemblages of GR and BS horizons are controlled by iron oxide, total organic carbon, nitrate, nitrite and sulfate. The BS microbiome guilds could potentially be regulated by higher nitrate but relatively lower nitrite and indiscriminately present (2 out of 6 samples) low sulfate levels. GR microbiome on the other hand is strongly affected by sulfate. Since we could not measure hydrogen and sulphide from the rock samples, their potential in influencing the microbial assemblages could not be ascertained in this analysis. Differential abundance of genes related to dissimilatory sulfate reduction (*dsr*B) and methanogenesis (*mcr*A) within BS and GR horizons possibly imply the disparity in recruitment of such processes within the subsurface provinces. These results suggest that microbial assemblages are affected by different geochemical parameters across the subsurface horizons of Deccan traps.

Microbial co-occurrence analysis indicates the presence of distinct and well defined networks across the three horizons, which corroborates the correlation based observation. Families affiliated to the first hotspot have previously been reported to be involved in diverse, yet deep biosphere relevant metabolic processes, *viz*., heterotrophic, anaerobic/microaerophilic fermentation, nitrate and sulfate reduction^87–92^. Nitrate and sulfate reducers of this group may be fuelled by hydrogen generated by the fermentative members. Members of the second hotspot are mainly anaerobes/facultative anaerobes, syntrophs, autotrophs, nitrate reducers, hydrogen- and iron oxidizers known to withstand different environmental stresses^21,93–97^. This hotspot represents microbial groups which are generally observed in extreme and oligotrophic environments and further highlight the link between autotrophic-fermentative/organotrophic-syntrophic organisms that could drive communities through anoxygenic carbon fixation, hydrogen metabolism, and nitrate/sulfate reduction. *Nitrospiracea*, *Cyanobacteria* and the other members of micro-network 1 play important roles in subsurface nitrogen and carbon cycle^98–101^, while *Thermoplasmataceae* and Terrestrial Miscellaneous Group archaea are known thermophiles, previously observed in hot springs and other igneous provinces^102^.

Microbial life in oligotrophic deep terrestrial subsurface hosted by igneous rocks are highly constrained by metabolic resources. Due to the extremely low cell turnover rates (hundreds to thousands of years), it is difficult to describe microbial processes by direct measurements^20^. Nevertheless, whole genome metagenome based studies, supported by 16S rRNA gene based analysis allow us to gain a better understanding. The metagenomic inventory supports our observation that there is a syntrophic relationship between autotrophic and heterotrophic organisms which could exchange fixed carbons, CO_2_, H_2_, etc. for their sustenance. Though there are presence of both denitrification and dissimilatory nitrate and sulfate reduction pathways for harvesting energy in the Deccan subsurface, nitrate appears to be the preferred energy source. Reduced sulfur, nitrite, iron and hydrogen could be potent electron donors to fuel the community metabolism. This inference is in line with previous reports on thermodynamics of microbial processes within deep biosphere as well as their metagenomic analyses^20,103^. The presence of different genes related to assimilatory as well as dissimilatory nitrogen metabolism indicates that there is likely a dynamic nitrogen cycling occurring within the Deccan subsurface. Overall abundance of *nir*B in all the samples (ammonia assimilation) is higher when compared to denitrification genes (*nir*K, *nir*S, *nor*B and *nos*Z). Results from previous studies indicate that ATP synthesis in denitrification is far lower than that expected from the free energy changes and even lower than in nitrate ammonification^104^. This justifies the fact that ammonification could be more common pathway when compared to denitrification in the Deccan subsurface not only for generating more ATP but also the product of ammonification (NH_3_) is more bioavailable than the product of denitrification, i.e., N_2_. Relative abundances of genes related to carbon cycle give a sense that CO_2_ could be the driving force in subsurface igneous provinces of Deccan traps. 3-hydroxypropionate pathway (3-HP) is found to be the most abundant carbon fixation pathway. In addition to CO_2_ fixation, this pathway enables synthesis of necessary metabolic intermediates and fatty acids from HCO_3_^−^ ^105^. Ample presence of HCO_3_^−^ in the igneous rocks including the Deccan traps, which contains highest HCO_3_^−^ concentration among other similar rock types and basaltic watersheds from around the world^106^, justifies the fact that 3-HP could be an important and common carbon fixation pathway. High abundance of HCO_3_^−^ in the Deccan traps selectively allows both chemoautotrophic and chemoorganotrophic organisms harbouring *acc*C to thrive in the Deccan subsurface. Similar result with ubiquity of *acc*C gene is obtained in deep Fennoscandian bed rock fluid samples^18^.

In view of the geochemical environment, taxonomic composition and metagenomic inventory, we infer that autotrophic carbon fixation, sulfur/hydrogen oxidation, fermentation and nitrification-denitrification are likely to be the key reactions driving the subsurface ecosystem linking the carbon, nitrogen and sulfur cycles. Products of fermentation and other organic molecules (generated through *acc*C activities) are used as potent substrates in the nutrient deprived subsurface provinces of Deccan traps. Low levels of organic molecules may be utilized by chemoorganotrophs for gaining energy or may be assimilated as a carbon source. CO_2_ is a useful fuel for the autotrophs which further produce nutrients for other heterotrophic community members. The coupling of redox transformation of sulfur and nitrogen substrates to carbon flux is clearly revealed from metagenomic data. Previous reports suggest that though organotrophic denitrification is more spontaneous than lithotrophic denitrification in presence of reduced sulfur (on the basis of ΔG values), presence of acetate may accelerate the process of denitrification and sulfur oxidation simultaneously^107,108^. Higher abundance of acetyl CoA synthase and acetate kinase among other genes related to fermentation in all the three metagenomes gives us a sense that the presence of acetate is prevalent in the Deccan subsurface. Though metagenomic binning would yield better results and provide improved understanding of the coupled metabolic processes, from the present scenario it can be hypothesized that sulfur oxidation coupled with denitrification in presence of acetate may be a common process in Deccan subsurface, considering the subsurface anaerobic environment of Deccan traps. Similar results have been reported in a 1.34 km deep fault zone of Witwatersrand Basin, South Africa^17^. Overall metagenomic data are in accordance with the taxonomic outcomes where close association of chemolithoautotrophs and heterotrophs are evident.

In conclusion, this is the first study that provides a comprehensive overview of the microbial community structure and their probable ecological role in the subterranean province of seismically active Koyna-Warna region in the Deccan traps. Our results reveal that the microbial communities are predominated by *Proteobacteria*, *Actinobacteria* and *Firmicutes*. Although less abundant, members of *Nitrospira*, *Spirochaetes*, *Chloroflexi*, *Epsilonproteobacteria* and *Cyanobacteria* could play key roles in driving the community function. Partitioning of interrelated microbial guilds on the basis of rock geochemistry suggests environment specific taxon recruitment. Overall, 16S rRNA gene based community composition supported by metagenomic inventory indicate that chemoautotrophic assimilation of carbon with oxidation of sulfur, hydrogen and nitrite coupled with fermentation and anaerobic respiration using nitrate as preferred terminal electron acceptor fuel the community. Limited methane metabolism coupled with sulfate reduction, fermentation or denitrification is also present. Interplay between chemolithoautotrophic and heterotrophic organisms and their metabolisms are highlighted.

## Methods

### Sample collection

Subsurface core samples were collected from different depths of three exploratory drill holes (Panchgani [P], Ukhalu [U] and Phansavale [PV]) in the Koyna-Warna region of Deccan traps, Maharashtra, India during July 2014 [Panchgani (N 17°18.112′, E 073°47.455′) and Ukhalu (N 17°07.552′, E 073°52.148′)] and May 2015 [Phansavale (N 17°09.017′, E 073°40.058′)] (Fig. 1). Samples were checked for the presence of sodium fluorescein (500 mg m^−3^) used during drilling according to the protocol of Nyyssönen *et al*.^16^. Only interior pieces of rock were selected for microbiological study to avoid potential drilling mud contamination as recommended elsewhere^109^. Samples were collected following aseptic techniques and stored in sterile containers at 4 °C for shipment. In laboratory, the samples were stored at −20 °C to limit the chance of microbial contamination, till further analysis.

### Rock processing

Thirteen rock core samples, four each from Panchgani and Phansavale, and five from Ukhalu were collected and processed for further study. Rock cores were washed thoroughly under sterile condition with autoclaved, DNA free water (Thermo-Fisher Scientific) and sub-coring of the rock was done to get rock samples devoid of any possible contamination that might have occurred during drilling. At first, the uneven ends of the cores were removed using granite cutter fitted with sterilized cutting blade. Subsequently, with either a mechanical drilling instrument fitted with sterilized drill bit or with sterilized chisel and hammer, rock powder was obtained from the core interior part. The rock powder thus obtained was stored in sterilized DNA free containers at −20 °C for further geochemical and microbial analysis.

### Geochemical analysis

Rock powder was sieved through 2 mm mesh. Acid digestion of the rock powders was carried out in a microwave digester (Milestone SK12) followed by the measurement of major elements by using ICP-MS (iCAP-Q, Thermo Scientific). Rock powders were ultra-sonicated in presence of water for major anions (Cl^−^, NO_2_^−^, SO_4_^2−^, NO_3_^−^,and PO_4_^3−^) analysis by using Dionex ICS-2100 (Thermo Scientific). Total organic carbon, total inorganic carbon and total carbon were measured on OI analytical TOC analyzer. Before analysing TOC, rock powders were fumigated with 1 N HCl for 48 hours under fume hood. Quantification of the elemental oxides was done using PANalytical Epsilon3 XRF instrument. All the measurements for major elements, anions, carbon content and oxides were carried out in triplicates and the standard error was <1% from the mean for all the analyses. Mineralogical analysis of the powdered rocks was conducted using PANalytical XRD X’pert Powder and on the basis of 2θ and d-spacing, mineral composition was evaluated using X’pert HighScore Plus software. Alkalinity of the rocks was measured using USEPA method 310.2. pH measurement was done by incubating 1 g of rock powder in 0.1 M CaCl_2_ at 1:10 ratio (w/v), and highly sensitive probes (Orion) fitted with an Orion multimeter (Thermo electron corporation, Beverly, MA) were used^110^.

### Extraction of metagenomic DNA

Metagenomic DNA was extracted from 13 samples using MoBio power soil DNA isolation Kit (MoBio) following the manufacturer protocol. Total DNA from reagent control was also extracted using the same procedure (as mentioned before) and were used subsequently to check any possible contamination. Quality of the extracted metagenomic DNA and its concentration was measured using NanoDrop 2000 spectrophotometer, followed by fluorometric quantitation using Qubit (Thermo-Fisher Scientific).

### Quantitative polymerase chain reaction (qPCR)

The bacterial and archaeal populations in a rock sample were quantified by estimating the copy number of bacterial and archaeal specific 16S rRNA gene. Copy numbers of two functional genes (*dsr*B and *mcr*A) were also quantified using qPCR-based technique to estimate the sulfate reducing and methanogenic populations. Details of qPCR primers used in the study are provided in Supplementary Table S4. 2 µl of metagenomic DNA was added to the PCR master-mix with a total volume of 10 µl. All the reactions were set up in triplicate. Quantitative PCR was performed in QuantStudio 5 with Power SYBR green PCR mastermix (Invitrogen), with primer concentration of 5 pM and the following amplification conditions: 95 °C for 10 minutes, 40 cycles of 95 °C for 15 sec, 55 °C for 30 sec and 72 °C for 30 sec. Melting curve analysis was run after each assay to check PCR specificity. Quantitative PCR was performed in triplicate and some of the samples having higher DNA yield were run in at least two dilutions to check for PCR inhibitions. Bacterial 16S rRNA gene copy numbers were determined in each sample by comparing the amplification result to a standard dilution series ranging from 10^2^ to 10^10^ of plasmid DNA containing the 16S rRNA gene of *Achromobacter* sp. MTCC 12117. Genes encoding archaeal 16S rRNA, *dsr*B, and *mcr*A were PCR amplified from the metagenome, cloned in TA cloning vector and plasmid DNAs for each with copy numbers 10^2^ to 10^10^ were used as standards for quantitation purpose.

### 16S rRNA gene amplification and sequencing

Following the quality check, metagenomes were subjected to amplification of V4 region of 16S rRNA gene using primers 515 F/806R^111^ (Supplementary Table S4). Each forward primer was tagged with sample specific 10–12 bp barcode for multiplexing during sequencing run. Duplicate PCR reactions were performed for each samples using AmpliTaq Gold 360 Mastermix (Thermo-Fisher Scientific) and the PCR products were gel purified using QIAquick Gel Extraction Kit (Qiagen). Concentrations of the purified PCR products were measured through Qubit and the purified amplicons from each sample were pooled with equimolar concentration and sequenced on Ion S5 platform (Thermo-Fisher Scientific). The sequence reads were submitted to short read archive under BioProject ID PRJNA389253.

### Quality filtering, operational taxonomic unit (OTU) picking and annotation

Raw data obtained from Ion S5 were preprocessed and quality filtered using the Quantitative Insights Into Microbial Ecology (QIIME 1.9.1)^112^ bioinformatics pipeline in which sequences having lengths outside bounds of 200 and 310, mean quality score below minimum of 25, maximum homopolymer run exceeding limit of 6 and number of mismatches in primer exceeding limit of 6 were filtered out. After filtering and removing potential erroneous reads, OTUs were picked for further analysis. De novo based clustering of reads to form OTU was performed using UCLUST under QIIME workflow. Sequences having greater than 97% similarity were assigned to same OTU. Representative reads from each OTU were assigned taxonomy using UCLUST trained SILVA 128 database^113^. OTUs present in the reagent control were removed from the OTU pool of the samples. Alpha diversity parameters were calculated using alpha_diversity.py under QIIME workflow. For comparable alpha diversity analysis, the data set was normalized by random sampling of 115812 reads per sample (i.e., lowest number of filtered reads detected across all the samples) and control OTUs (i.e., OTUs present in reagent control) were removed and alpha diversity parameters were calculated as mentioned before. Average abundance of each phylum was calculated considering values obtained for samples from each horizon.

### Statistical analysis

Principal component analysis (PCA) was performed using PAST software^114^ on the basis of geochemical parameters. Geochemical parameters were normalized on the basis of feature scaling before PCA was plotted. Statistical significance of similarity of geochemical parameters across different horizons was evaluated on Euclidean matrix in PAST software using analysis of similarities (ANOSIM). Average abundances of each phylum across all the samples were calculated and compared. Standard deviations (SD) were calculated during average calculations and mentioned in brackets. Classes having average abundance of 0.1% across all the samples were selected for correlation analysis and correlation heatmap was constructed on the basis of Spearman correlation using METAGENassist^115^. Distance based redundancy analysis (db-RDA) of Bray-Curtis distance on the basis of all the microbial classes present across 11 samples, and 10 different physicochemical parameters was performed using Vegan package in R statistical software^116^. Similarity percentage (SIMPER) analysis was also done using PAST software for 11 samples to understand the microbial classes responsible for the grouping of different rock types. Two samples, PV2 and PV8 were not considered for db-RDA and SIMPER analysis due to over-representation of a single OTU in these samples. OTU overlap among the samples from similar rock type and among different rock types were elucidated using InteractiVenn^117^ and Venny 2.1^118^.

### Network analysis

Co-occurrence network analysis was performed considering the microbial families detected in the samples. Microbial families having greater than 0.1% average abundance across all the samples was used for the network construction. Correlation among different families was calculated using otu.association command in mothur^119^. Based on pairwise Spearman correlations with correlation values >0.75 and <−0.75 were used for construction of co-occurrence network using Cytoscape 3.4.0^120^.

### Whole metagenome sequencing and analysis

Metagenomes of three representative samples from basaltic (U7), transition (U6) and granitic zones (U11) were considered for whole metagenome sequencing. After multiple DNA extraction, concentrations of DNA were determined by Quant-iT Picogreen dsDNA assay. DNA samples were fragmented to ~250 base pairs (bp) using a Covaris S220 Focused-ultrasonicator (Covaris Inc. Woburn, MA) and paired end (2 × 151 bp) sequencing was performed at Marine Biological laboratory, Woods Hole, USA using Illumina NextSeq500 platform under Deep Carbon Observatory (DCO), Census of Deep Life program. Details of metagenomic library preparation and sequencing are mentioned in the supplementary information. The raw sequences were adapter trimmed using cutadapt 1.9.1^121^. Quality checking of the trimmed paired end reads was conducted using FastQC (Version 0.11.7). Trimmed sequences were assembled using metaSPAdes assembler^122,123^ using default parameters. Quast 4.3 was used for evaluations of metagenomic assemblies and different assembly statistics^124^. K-mer of 55 was chosen (on the basis of longest contig) for annotation using Genomes OnLine Database (GOLD) v.6^125^. KEGG based annotation was selected for further analysis and metabolic pathway reconstruction was done. The annotated data are available under IMG GOLD Analysis Project ID Ga0232666, Ga0232668 and Ga0232669.

## Electronic supplementary material

Supplementary Information
