# Supplementary material for: Exploration of deep terrestrial subsurface microbiome in Late Cretaceous Deccan traps and underlying Archean basement, India
Source: Sci Rep. 2018 Nov 29;8:17459. doi: 10.1038/s41598-018-35940-0 (PMC6265293; doi:10.1038/s41598-018-35940-0)
Supplement: Supplementary file 1 — Supplementary Information [file 41598_2018_35940_MOESM1_ESM.pdf]

Exploration of deep terrestrial subsurface microbiome in Late Cretaceous Deccan traps and underlying Archean basement, India

Avishek Dutta[1,2], Srimanti Dutta Gupta[3], Abhishek Gupta[1], Jayeeta Sarkar[1], Sukanta Roy[4,5], Abhijit Mukherjee[3,6], *Pinaki Sar[1]

[1]Environmental microbiology and genomics laboratory, Department of Biotechnology, Indian Institute of Technology Kharagpur, Kharagpur, 721302

[2]School of Bioscience, Indian Institute of Technology Kharagpur, Kharagpur, 721302

[3]School of Environmental Science and Engineering, Indian Institute of Technology Kharagpur, Kharagpur, 721302

[4]Ministry of Earth Sciences, Borehole Geophysics Research Laboratory, Karad, 415114

[5]CSIR-National Geophysical Research Institute, Hyderabad, 500007

[6]Department of Geology and Geophysics, Indian Institute of Technology Kharagpur, Kharagpur, 721302

Corresponding author:

Pinaki Sar

Email ID: sarpinaki@yahoo.com; psar@bt.iitkgp.ac.in

(a) Scientific Borehole location: Phansavale

Latitude: N17° 09.017' **Longitude:** E073° 40.058' **Altitude**=131 m

Sample Name	Depth (meters below surface)	Nature
PV4	106.4	Basalt
PV2	322.41	Basalt
PV6	500.7	Transition zone
PV8	521.8	Granite

(b) Scientific Borehole location: Ukhalu

Latitude: N 17° 07.552' **Longitude:** E 073° 52.148' **Altitude**=567 m

Ukhalu	Depth (meters below surface)	Nature
U9	59.6	Massive Basalt
U8	262.15	Amygdaloidal Basalt
U7	456.7	Basalt
U6	781.92	Transition zone
U11	1365.48	Granite

(c) Scientific Borehole location: Panchgani

Latitude: N 17° 18.112' **Longitude:** E 073° 47.455' **Altitude**=960 m

Panchghani	Depth (meters below surface)	Nature
P3	1241.2	Basalt
P2	1254.19	Transition zone
P4	1299.89	Granite
P1	1490	Granite

Table S1. List of samples recovered from different scientific boreholes **(a).** Samples recovered from borehole at Phansavale **(b).** Samples recovered from borehole at Ukhalu **(c).** Samples recovered from borehole at Panchgani

		U9	U8	U7	PV4	PV2	U6	P3	P2	P4	PV6	PV8	P1	U11
		**Basalt**	**Basalt**	**Basalt**	**Basalt**	**Basalt**	**Transition**	**Basalt**	**Transition**	**Granite**	**Transition**	**Granite**	**Granite**	**Granite**
	**Normalized Depth (m)**	507.4	304.9	110.3	24.6	-191.4	-214.9	-281.2	-294.2	-339.89	-369.7	-390.8	-530.0	-798.5
	**Actual Depth (m)**	59.6	262.2	456.7	106.4	322.4	781.9	1241.2	1254.2	1300.01	500.7	521.8	1490.0	1365.4
**Elements (mg/kg)**	**Cd**	1.4	0.8	4.8	1.8	2.3	4.7	4.4	3.8	11.3	3.5	10.0	13.3	15.0
	**Ca**	3046.6	4044.0	5117.4	6427.5	6481.4	8948.9	9982.3	10667.3	9031.0	8204.2	8282.9	13547.3	9952.5
	**Cr**	121.3	120.1	105.0	165.7	180.9	135.2	330.1	345.8	330.0	256.3	480.8	495.8	240.7
	**Fe**	1875.8	1845.7	1650.7	2866.3	2925.7	2115.7	3000.8	3211.3	2580.0	3060.7	3945.7	3870.9	2761.4
	**K**	3886.5	3945.7	3811.5	7711.2	7816.5	4711.3	8175.7	9137.3	6239.6	7942.5	8220.1	9360.2	4546.7
	**Mg**	6195.7	4575.7	5087.2	4546.0	4890.8	6060.8	5388.1	6750.7	5990.0	6225.8	7800.8	8985.9	7440.4
	**Na**	4995.7	4860.9	4951.0	4410.4	3976.3	4845.1	4830.7	4651.3	3020.0	3345.7	3060.9	4530.8	4666.0
**Oxides (%)**	**Al_2O_3**	9.6	9.7	9.6	6.9	9.3	13.7	10.0	11.6	13.6	12.9	12.3	10.7	12.4
	**Na_2O**	0.2	0.9	0.2	0.6	2.1	4.3	1.0	5.6	1.0	2.2	5.0	0.7	0.9
	**MnO**	0.3	0.3	0.3	0.3	0.3	0.1	0.3	0.0	0.1	0.0	0.1	0.4	0.1
	**MgO**	1.9	2.0	1.6	2.8	1.5	1.8	2.3	0.9	0.3	1.9	2.0	4.1	4.4
	**SiO_2**	34.4	34.9	35.0	35.6	40.4	52.8	33.6	65.1	70.1	55.6	59.8	44.2	53.7
	**K_2O**	0.1	0.3	0.2	0.4	1.0	2.0	0.2	2.2	5.7	0.9	1.9	0.4	0.6
	**CaO**	16.0	12.3	14.0	10.3	10.8	4.7	14.1	4.3	5.1	5.1	3.7	14.7	3.4
	**TiO_2**	3.9	3.6	4.5	4.9	3.4	2.0	4.5	1.0	0.7	1.7	1.1	1.4	2.1
	**V_2O_5**	0.1	0.1	0.1	0.1	0.1	0.0	0.1	0.0	0.0	0.0	0.0	0.1	0.3
	**Fe_2O_3**	31.7	34.0	32.6	35.9	29.7	17.0	32.0	7.6	3.2	19.2	12.7	20.4	18.0
**Carbon (mg/kg)**	**TOC**	48.1	46.0	25.2	34.2	31.0	20.4	23.8	22.4	12.7	20.8	9.3	12.6	10.2
	**TIC**	98.3	93.2	96.5	101.2	95.1	89.5	98.2	93.5	39.6	88.2	49.1	62.5	59.5
	**TC**	149.3	143.0	129.4	137.2	124.0	109.8	122.1	115.0	50.3	104.5	61.7	76.2	70.0
**Anions (mg/kg)**	**Cl^-**	230.7	109.0	369.9	73.8	75.1	72.6	75.9	376.9	159.3	124.0	107.4	283.1	700.8
	**NO_2^-**	18.4	0.3	2.5	11.4	0.6	12.6	17.2	11.5	1.8	2.7	14.2	2.9	0.2
	**SO_4^{2-}**	0.0	834.5	885.3	0.0	0.0	528.1	0.0	1572.4	1699.2	0.0	978.7	1781.7	1001.6
	**NO_3^-**	1.4	67.0	0.0	22.8	0.0	0.0	0.0	0.0	14.3	0.0	53.1	12.2	47.4
	**PO_4^{3-}**	0.0	0.0	0.0	0.0	0.0	0.0	0.0	0.0	0.0	0.0	19.2	21.5	57.3
**Others**	**Alkalinity (mg/kg)**	58.5	19.5	97.2	19.5	19.5	136.3	97.6	643.8	895.7	175.4	948.8	989.1	219.8
	**pH**	6.8	6.8	7.5	6.7	6.8	7.4	6.4	6.9	7.8	7.2	7.9	8.9	10.2

Table S2. Geochemistry of the samples from three different subsurface horizons of Deccan traps.

Assembly	U7(BS)	U6(TZ)	U11(GR)
**Raw bases sequenced (bp)**	5,177,929,222	4,425,392,334	4,785,727,862
**Filtered bases for assembly(bp)**	5,129,509,685	4,388,109,357	4,730,161,325
**# contigs (>= 0 bp)**	3551184	3043517	3079165
**# contigs (>= 1000 bp)**	59341	24100	21234
**# contigs (>= 5000 bp)**	2489	1108	1801
**# contigs (>= 10000 bp)**	817	439	871
**# contigs (>= 25000 bp)**	240	175	301
**# contigs (>= 50000 bp)**	83	68	99
**Total length (>= 0 bp)**	1121342323	904785706	928081375
**Total length (>= 1000 bp)**	129668997	56432465	62148129
**Total length (>= 5000 bp)**	32708051	18967914	30754754
**Total length (>= 10000 bp)**	21425979	14462790	24269804
**Total length (>= 25000 bp)**	12713431	10511917	15665541
**Total length (>= 50000 bp)**	7399723	6820289	8782700
**Largest contig**	288431	406716	336897
**Total length**	252255710	145070959	159745178
**GC (%)**	50.48	48.97	48.3
**N50**	1035	782	770
**N75**	641	587	585
**L50**	55859	42534	41805
**L75**	136139	97241	102567
**Percentage abundance (Archaea) COG 30 % and above**	0.063251995	0.051657573	0.049272766
**Percentage abundance (Bacteria) COG 30 % and above**	40.16661168	38.34491192	34.05431934
**Percentage abundance (Eukaryotes) COG 30 % and above**	5.029558728	5.048523556	5.203950939
**Percentage abundance (Viruses) COG 30 % and above**	0.151637737	0.153104452	0.127692119
**Unassigned**	54.58893986	56.4018025	60.56476483
**Percentage archaea with respect to prokaryotes**	0.157226472	0.134536948	0.144479695
**Percentage bacteria with respect to prokaryotes**	99.84277353	99.86546305	99.8555203

Table S3. Metagenomic sequencing information and assembly statistics of representative samples from different horizons of Deccan traps

Gene	Primer Name	Primer Sequence (5'-3')	Source
Bacterial 16S rRNA gene (qPCR)	P1	CCTACGGGAGGCAGCAG	Muyzer et al., 1993[1]
	P2	ATTACCGCGGCTGCTGG	
Archaeal 16S rRNA gene (qPCR)	A344f	ACGGGGCGCAGCAGGCGCGA	Bano et al., 2004[2]
	Ar774r	CCCGGGTATCTAATCC	Barns et al. 1994[3]
*dsr*B (qPCR)	DSRp2060F	CAACATCGTYCAYACCCAGGG	Geets et al., 2006[4]
	DSR4R	GTGTAGCAGTTACCGCA	Wagner et al., 1998[5]
*mcr*A (qPCR)	ME1	GCMATGCARATHGGWATGTC	Hales et al., 1996[6]
	ME3	TGTGTGAAWCCKACDCCACC	Nyyssönen et al., 2012[7]
Bacterial 16S rRNA gene (Amplicon sequencing)	F515	GTGCCAGCMGCCGCGGTAA	Bates et al., 2011[8]
	R806	GGACTACVSGGGTATCTAAT	

Table S4. Quantitative PCR and amplicon sequencing primer details

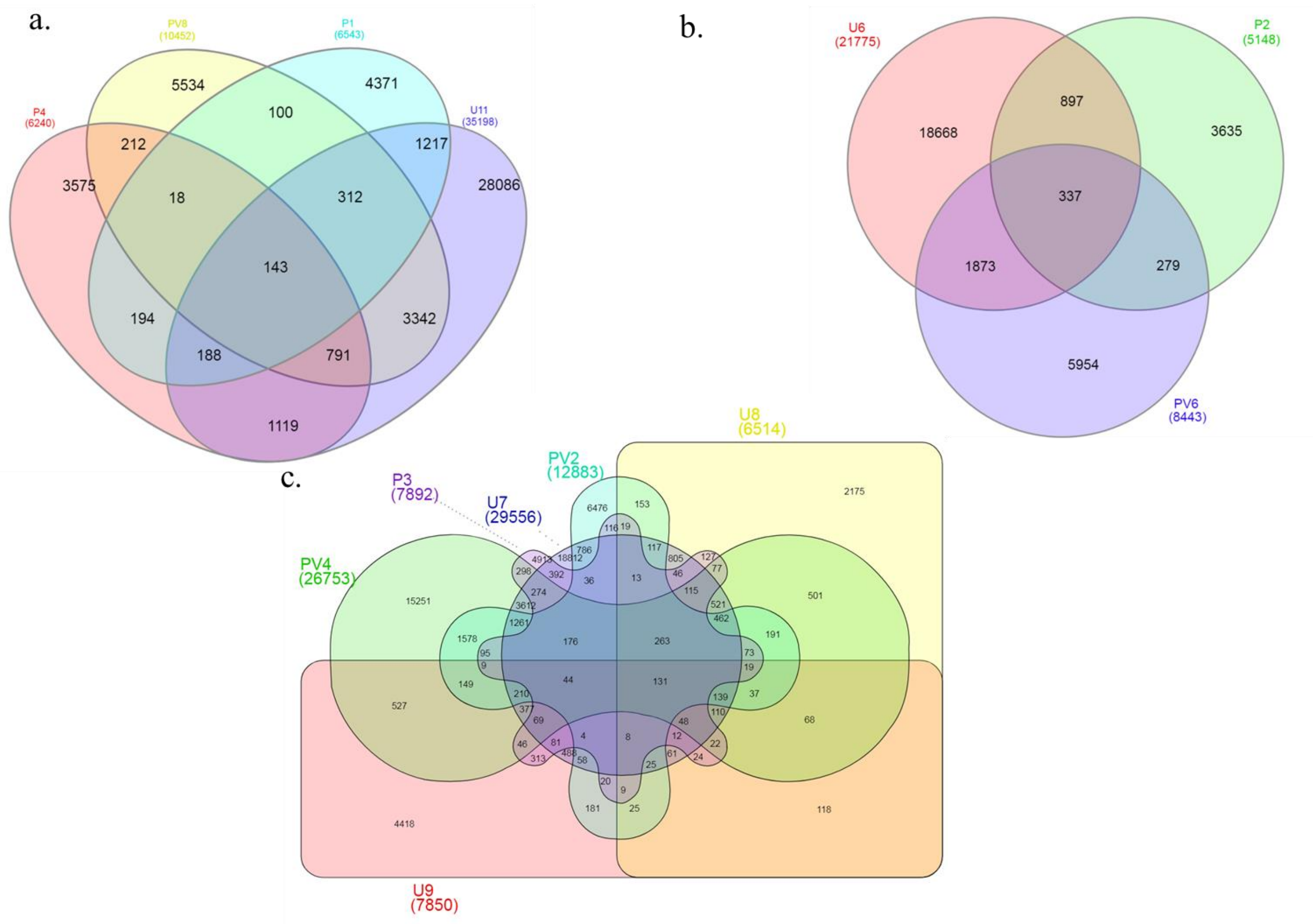

Figure S1. Venn diagram of OTUs present in (a) granitic (b) transition and (c) basaltic zones. OTUs were based on 97% sequence similarity. The numbers inside the diagram are the number of OTUs.

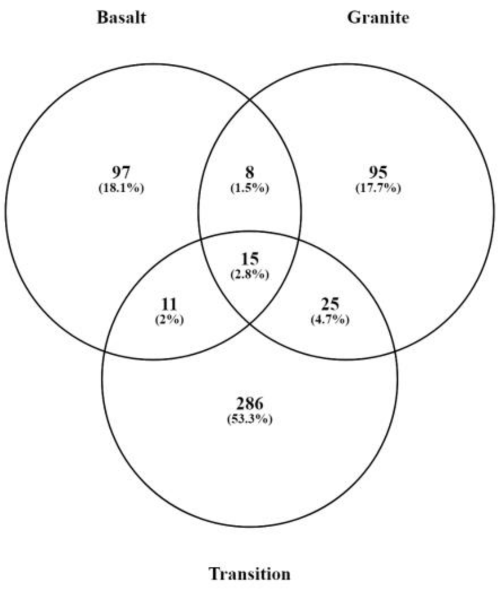

Figure S2. Venn diagram of the core OTUs present in different subsurface horizons of Deccan traps

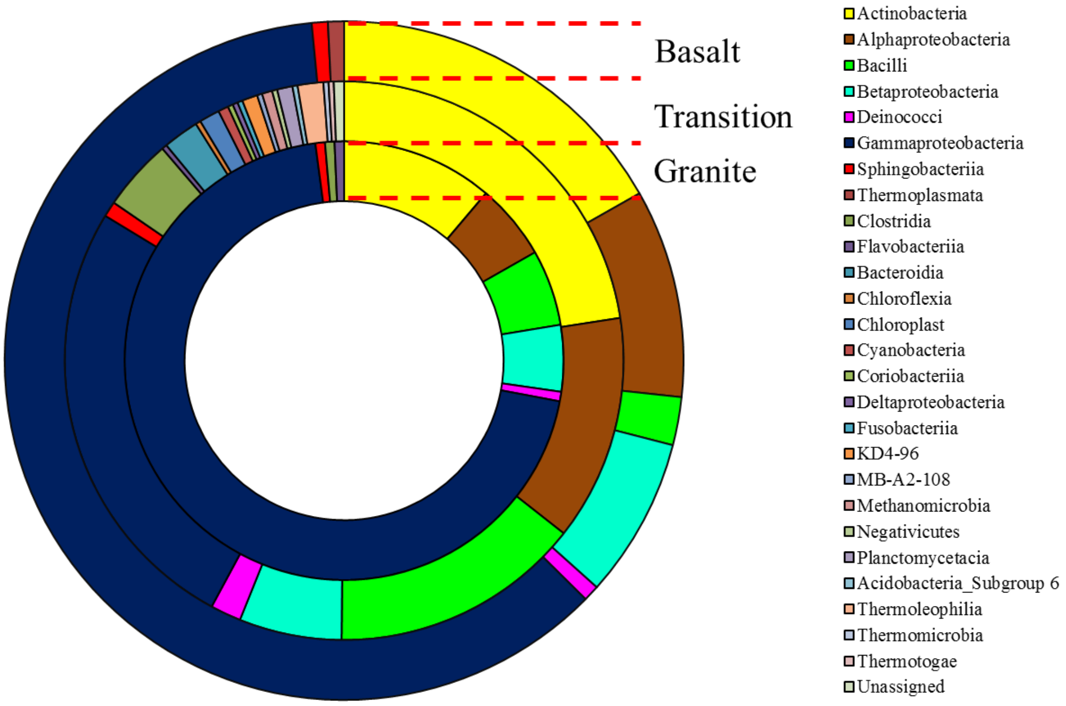

Figure S3. Taxonomic distribution of number of core OTUs across three different horizons of Deccan traps

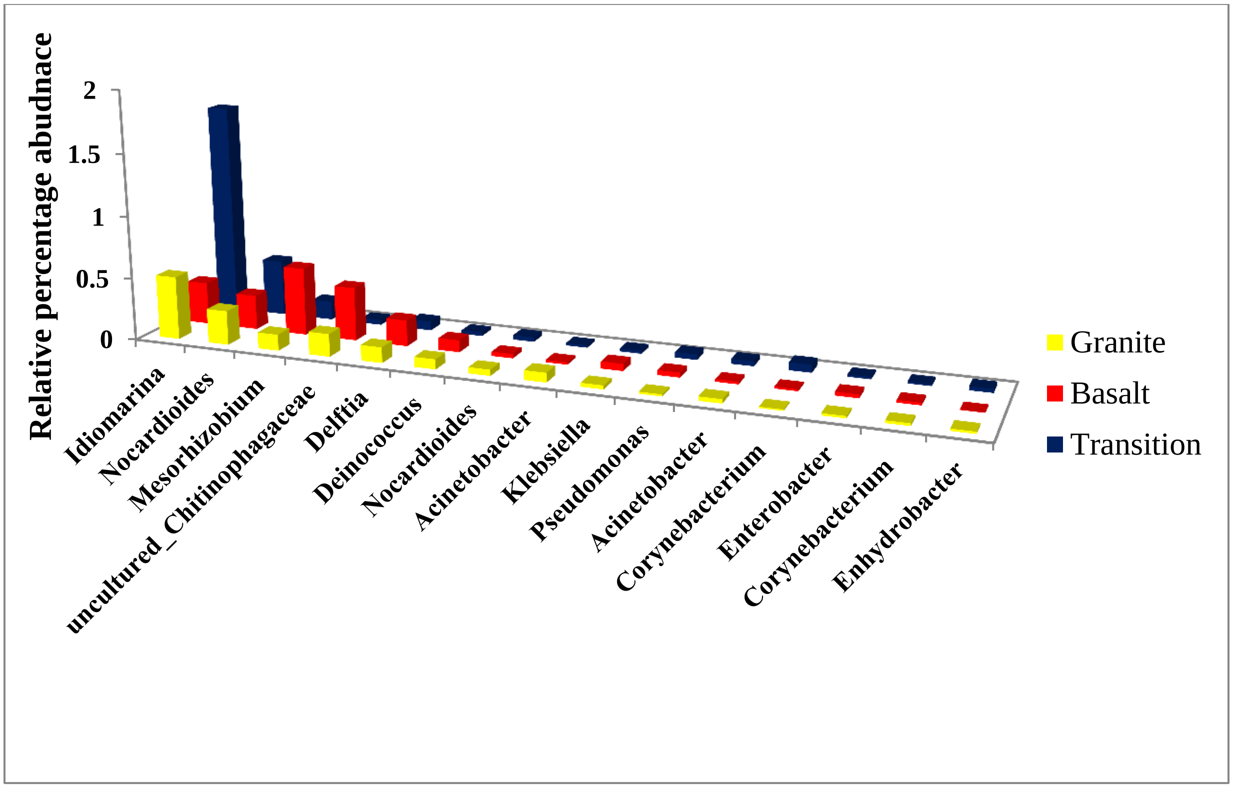

Figure S4. Rank abundance histogram for shared OTUs across three different rock types

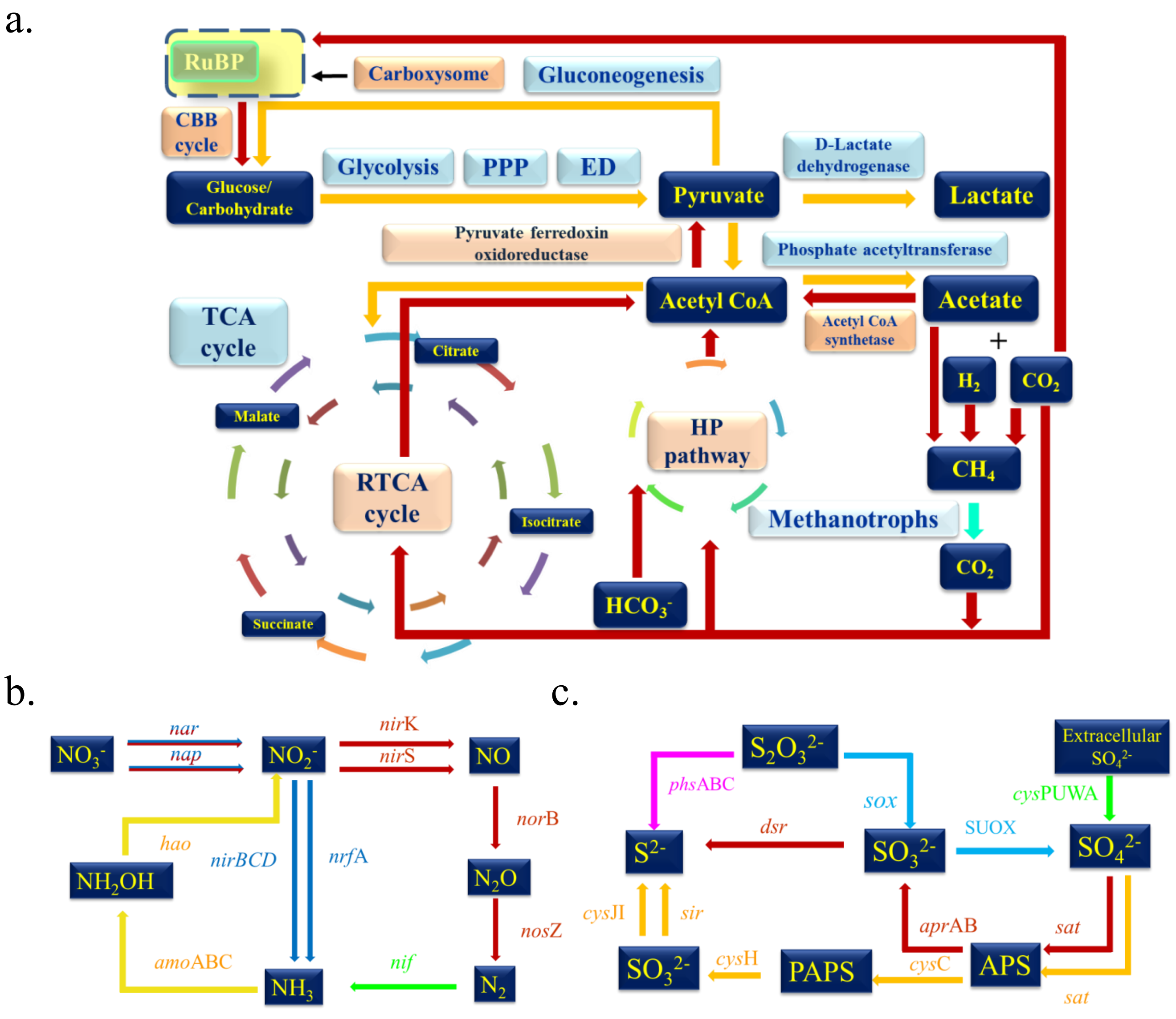

Figure S5 (a). Genes detected in subsurface metagenome and possible pathways present in C cycle. Red colour represents pathways for carbon fixation; Yellow colour represents other major pathways of carbon metabolism. **(b)**. Genes detected in subsurface metagenome and possible pathways present in N cycle. Blue colour represents gene taking part in ammonification (respiration/assimilation); Red colour represents gene taking part in denitrification; Green colour represents gene taking part in nitrogen fixation ; Yellow colour represents gene taking part in nitrification. **(c).** Genes detected in subsurface metagenome and possible pathways present in S cycle. Blue colour represents gene taking part in sulfur oxidation; Red colour represents gene taking part in dissimilatory sulfate reduction; Green colour represents gene taking part sulfate transport; Yellow colour represents gene taking part in assimilatory sulfate reduction.

Methods

Metagenomic library preparation and sequencing

The concentrations were determined by Quant-iT Picogreen dsDNA assay (Life Technologies, Carlsbad, CA). Samples were fragmented to ~250 base pairs (bp) using a Covaris S220 Focused-ultrasonicator (Covaris Inc. Woburn, MA). Metagenomic libraries were prepared according to the Nugen Ovation® Ultralow Library system protocol (NuGen Technologies, Inc. San Carlos, CA). Libraries were visualized on an Agilent DNA 1000 Bioanalyzer chip (Agilent Technologies, Santa Clara, CA) and quantified using a KAPA SYBR ® FAST Universal qPCR Kit (KAPA Biosystems, Boston, MA). Paired-end sequencing (2 × 151 bp) was performed on an Illumina NextSeq 500 (Illumina, Inc. San Diego, CA). Base calls and quality scores were generated using Real-Time Analysis v2 software (Illumina, Inc. San Diego, CA) on the NextSeq 500 instrument, while sample demultiplexing and individual FASTQ files were completed utilizing a bcl2fastq Conversion Software v1.84 (Illumina Inc. San Diego, CA).

References

1. Muyzer, G., De Waal, E. C. & Uitterlinden, A. G. Profiling of complex microbial populations by denaturing gradient gel electrophoresis analysis of polymerase chain reaction-amplified genes coding for 16S rRNA. *Appl. Environ. Microbiol.* **59,** 695–700 (1993).

2. Bano, N., Ruffin, S., Ransom, B. & Hollibaugh, J. T. Phylogenetic composition of Arctic Ocean archaeal assemblages and comparison with Antarctic assemblages. *Appl. Environ. Microbiol.* **70,** 781–789 (2004).

3. Barns, S. M., Fundyga, R. E., Jeffries, M. W. & Pace, N. R. Remarkable archaeal diversity detected in a Yellowstone National Park hot spring environment. *Proc. Natl. Acad. Sci.* **91,** 1609–1613 (1994).

4. Geets, J. *et al.* DsrB gene-based DGGE for community and diversity surveys of sulfate-reducing bacteria. *J. Microbiol. Methods* **66,** 194–205 (2006).

5. Wagner, M., Roger, A. J., Flax, J. L., Brusseau, G. A. & Stahl, D. A. Phylogeny of dissimilatory sulfite reductases supports an early origin of sulfate respiration. *J. Bacteriol.* **180,** 2975–2982 (1998).

6. Hales, B. A. *et al.* Isolation and identification of methanogen-specific DNA from blanket bog peat by PCR amplification and sequence analysis. *Appl. Environ. Microbiol.* **62,** 668–675 (1996).

7. Nyyssönen, M. *et al.* Methanogenic and sulphate-reducing microbial communities in deep groundwater of crystalline rock fractures in Olkiluoto, Finland. *Geomicrobiol. J.* **29,** 863–878 (2012).

8. Bates, S. T. *et al.* Examining the global distribution of dominant archaeal populations in soil. *ISME J.* **5,** 908–917 (2011).
